# Respiratory loss during late-growing season determines the net carbon dioxide sink in northern permafrost regions

**DOI:** 10.1038/s41467-022-33293-x

**Published:** 2022-09-26

**Authors:** Zhihua Liu, John S. Kimball, Ashley P. Ballantyne, Nicholas C. Parazoo, Wen J. Wang, Ana Bastos, Nima Madani, Susan M. Natali, Jennifer D. Watts, Brendan M. Rogers, Philippe Ciais, Kailiang Yu, Anna-Maria Virkkala, Frederic Chevallier, Wouter Peters, Prabir K. Patra, Naveen Chandra

**Affiliations:** 1 Numerical Terradynamic Simulation Group, WA Franke College of Forestry and Conservation, University of Montana, Missoula, MT USA; 2 CAS Key Laboratory of Forest Ecology and Management, Institute of Applied Ecology, Chinese Academy of Sciences, Shenyang, Liaoning China; 3 Department of Ecosystem and Conservation Sciences, University of Montana, Missoula, MT USA; 4 Laboratoire des Sciences du Climat et de l’Environnement, LSCE/IPSL, CEA-CNRS-UVSQ, Université Paris-Saclay, Gif-sur-Yvette, France; 5 Jet Propulsion Laboratory, California Institute of Technology, Pasadena, CA USA; 6 Northeast Institute of Geography and Agroecology, Chinese Academy of Sciences, Changchun, Changchun, Jilin China; 7 Max Planck Institute for Biogeochemistry, Department of Biogeochemical Integration, Jena, Germany; 8 Woodwell Climate Research Center, Falmouth, MA USA; 9 Meteorology and Air Quality Group, Wageningen University and Research, Wageningen, the Netherlands; 10 Research Institute for Global Change, Japan Agency for Marine‐Earth Science and Technology (JAMSTEC), Yokohama, Japan; 11 University, Centre for Isotope Research, Groningen, the Netherlands

**Keywords:** Carbon cycle, Carbon cycle, Climate sciences

## Abstract

Warming of northern high latitude regions (NHL, > 50 °N) has increased both photosynthesis and respiration which results in considerable uncertainty regarding the net carbon dioxide (CO_2_) balance of NHL ecosystems. Using estimates constrained from atmospheric observations from 1980 to 2017, we find that the increasing trends of net CO_2_ uptake in the early-growing season are of similar magnitude across the tree cover gradient in the NHL. However, the trend of respiratory CO_2_ loss during late-growing season increases significantly with increasing tree cover, offsetting a larger fraction of photosynthetic CO_2_ uptake, and thus resulting in a slower rate of increasing annual net CO_2_ uptake in areas with higher tree cover, especially in central and southern boreal forest regions. The magnitude of this seasonal compensation effect explains the difference in net CO_2_ uptake trends along the NHL vegetation- permafrost gradient. Such seasonal compensation dynamics are not captured by dynamic global vegetation models, which simulate weaker respiration control on carbon exchange during the late-growing season, and thus calls into question projections of increasing net CO_2_ uptake as high latitude ecosystems respond to warming climate conditions.

## Abstract (web summary)

The northern high latitude permafrost region has been an important contributor to the carbon sink since the 1980s. A new study finds that as tree cover increases, respiratory CO2 loss during late-growing season offsets photosynthetic CO2 uptake and leads to a slower rate of increasing annual net CO2 uptake.

## Introduction

The northern high latitudes (NHL, > 50°N) are experiencing dramatic changes in carbon cycling, evidenced by an increase in the annual terrestrial net CO_2_ uptake and in the amplitude of the seasonal cycles of atmospheric CO_2_ over the past five decades^1–3^, but the mechanisms underlying these changes remain highly uncertain. Net CO_2_ uptake results from the imbalance between the much larger gross fluxes of plant photosynthesis and ecosystem respiration, which have asynchronous responses to seasonal climatic and environmental change^4–6^. For example, increased plant photosynthetic CO_2_ fixation during the growing season^7,8^ may be offset by enhanced respiratory CO_2_ release in the fall and/or winter^9,10^. Such offsets in net CO_2_ uptake among seasons (i.e., seasonal compensation) complicates the detection of the climate-carbon feedbacks at longer time scales over NHL ecosystems^4,11^. Further, the seasonal compensation in CO_2_ uptake may vary among biomes given the different sensitivity of above- and belowground carbon cycle processes to climate and environmental controls over changing NHL permafrost conditions^12–14^. Therefore, understanding the magnitude, trends, and spatial patterns of seasonal net CO_2_ uptake and its underlying mechanisms is important to address a fundamental question of whether net CO_2_ exchange has changed over the NHL, especially in rapidly changing permafrost regions^15–17^.

To gain insight into the trends and mechanisms of seasonal CO_2_ exchange in the NHL, we addressed the following three major questions. First, what are the trends in net CO_2_ uptake and how do they relate to climate, vegetation, and environmental gradients across the NHL? To answer this question, we analyzed an ensemble mean of long-term atmospheric CO_2_ inversions (ACIs, *n* = 6, 1980–2017) and a network of Eddy Covariance (EC) observations, each with at least three years of continuous measurements (*n* = 48 sites, 426 site-years, 1990–2017, Fig. S1, Table S1). Second, what are the mechanisms underlying different net CO_2_ uptake trends in the NHL? We hypothesized that the different net CO_2_ uptake trends were driven by: (H1) different temperature sensitivities of vegetation primary productivity or (H2) the degree to which seasonal net CO_2_ uptake is compensated by seasonal respiration losses. We then used structural equation modeling to explore climatic, environmental and vegetation controls on seasonal CO_2_ dynamics. Lastly, how well do the latest land surface models replicate the NHL seasonal CO_2_ dynamics? To answer this question, we compared the observationally constrained estimates with an ensemble (*n* = 10, 1980–2017) of Dynamic Global Vegetation Models (DGVMs) from the TRENDY intercomparison project (see Methods).

Here, we show that positive trends of annual net CO_2_ uptake are smaller with increasing tree cover. We attribute this pattern to the increased magnitude of seasonal compensation due to larger respiratory CO_2_ losses during late-growing season at greater levels of tree cover, rather than different temperature sensitivities of vegetation productivity across the NHL. Our synthesis investigation of extensive and diverse empirical datasets shows no evidence that NHL permafrost regions have become net sources of CO_2_ to the atmosphere; rather, we find that NHL permafrost regions appear to be gaining C through complex interactions between above and belowground processes that respond differently to seasonal climate. However, current DGVMs are unable to reproduce the different trends of net CO_2_ uptake along permafrost-vegetation gradients, potentially calling into question current projections of CO_2_ dynamics in the NHL.

## Results

### Trend of net CO_2_ uptake is strongly associated with tree cover in the NHL

**Fig. 1:**
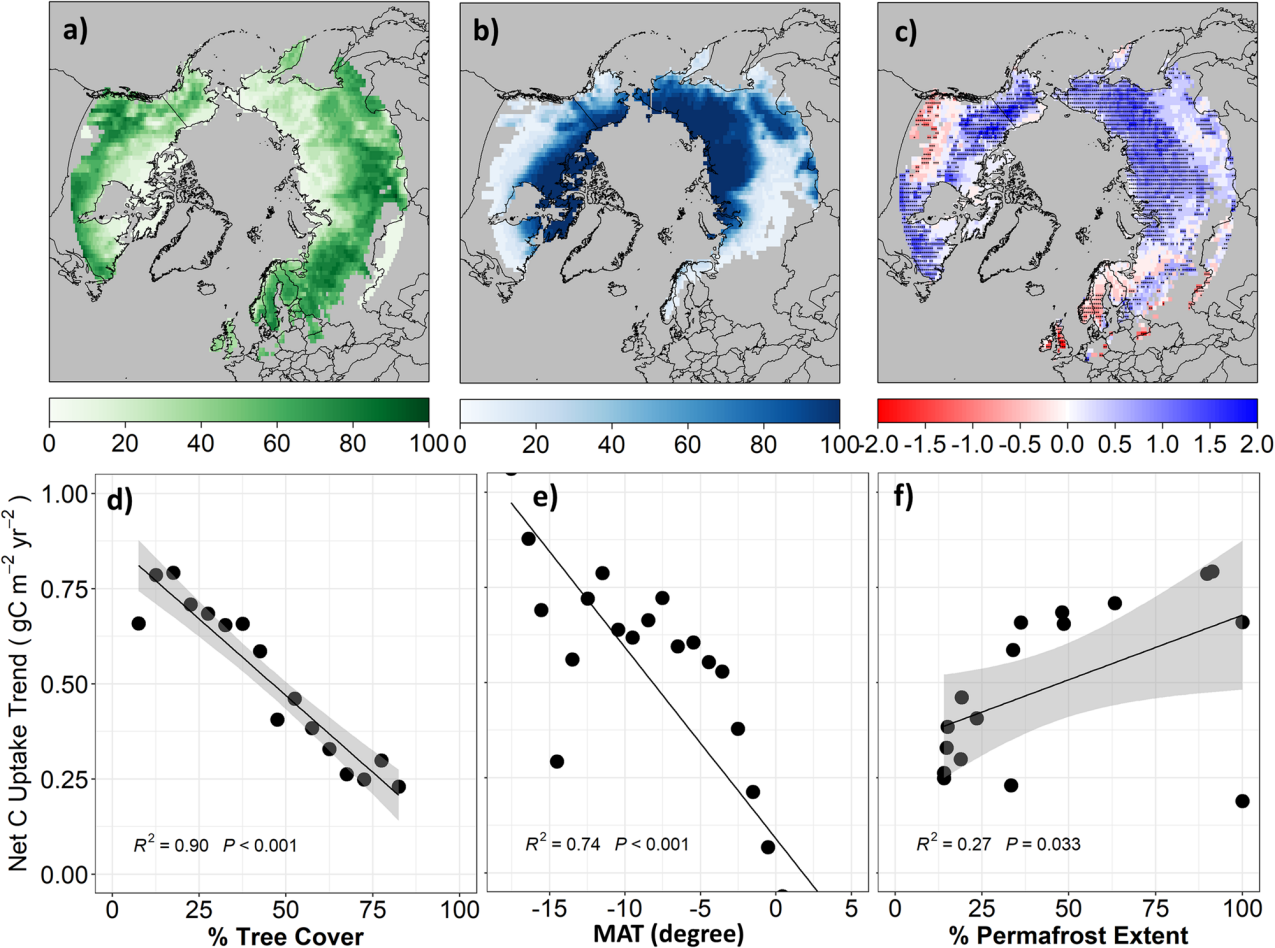
Net CO_2_ uptake is increasing faster in regions of lower tree cover and greater permafrost extent from 1980 to 2017 across the NHL ( > 50°N) according to an ensemble of ACIs. The maps show regional patterns of **a** percent tree cover (%TC) based on 35-year (1982-2016) TC layers observed by the Advanced Very High-Resolution Radiometer (AVHRR)^42^; **b** percent of permafrost extent (%P) based on the average over 1997–2017 from European Space Agency (ESA) Climate Change Initiative (CCI) Permafrost project data. **c** The annual trend of net CO_2_ uptake (gC m^−2^ yr^−2^) from 1980 to 2017 from the ensemble mean of six ACIs in the NHL. Points indicate trends are significant at 0.05 level. **d**–**f** Correlation between trends of net CO_2_ uptake with tree cover, mean annual temperature (MAT), and permafrost extent, respectively.

The ensemble mean of NHL ACIs (inversions) is highly correlated (r = 0.78, *p* < 0.001) with independent estimates of net CO_2_ exchange at the global scale (Fig. S2)^18^, thus giving us confidence to detect regional trends of net CO_2_ uptake emerging from the ‘noise’ associated with individual model errors and uncertainties. Estimates from ACIs showed that the NHL contributed roughly 28.7% of the mean global land CO_2_ sink (0.67 ± 0.28 PgC yr^−1^, where positive values denote net C uptake), 17% of the respective global trend (10.4 + 2.02 TgC yr^−2^), and 12.5% of the interannual variability in the global land sink, consistent with another recent model-based assessment^19^. From 1980 to 2017, about half (50.3%) of the NHL showed significant (*p* < 0.05) increases in annual net CO_2_ uptake, mostly in the permafrost region dominated by tundra shrub and graminoids (Fig. 1a–c, Fig. S1). In contrast, only 4.6% of the NHL showed significant (*p* < 0.05) decreases in annual net CO_2_ uptake, mostly in non-permafrost regions dominated by forest (Fig. 1a–c, Fig. S1). The trend of net CO_2_ uptake most strongly correlated with tree cover (TC, R^2^ = 0.90, *p* < 0.001), followed by mean annual temperature (R^2^ = 0.77, *p* < 0.001) and permafrost extent (P, R^2^ = 0.27, *p* = 0.033). This suggests that the distribution of forest vegetation, broadly shaped by climate and environmental conditions, is a primary control on net CO_2_ uptake trends in the NHL. Despite the fact that net CO_2_ uptake in forested regions increased at a slower rate, those regions are still stronger CO_2_ sinks than non-forest tundra in colder permafrost regions (Fig. S3). CO_2_ source regions are mainly located in the tundra of Alaska and Northeast Siberia, similar to patterns shown by recent analyses^10,20^, but the net CO_2_ source area is shrinking due to a strong positive trend in net CO_2_ uptake (Fig. S3).

To reduce the pixel-level uncertainty in estimating net CO_2_ uptake using ACIs, we aggregated net CO_2_ uptake to larger regions based on tree cover and permafrost extent, which are negatively correlated in the NHL (R = −0.79, *p* < 0.001, Fig. S4). Regional aggregation of the trends showed that net CO_2_ uptake is increasing at a significantly faster rate in tundra (TC < 50%) permafrost (*P* > 10%) regions than in tree-dominated (TC > 50%) non-permafrost regions (*P* < 10%). However, the trends of net CO_2_ uptake are not statistically different between low tree cover (TC < 30%) in continuous (*P* > 90%) permafrost regions and intermediate tree cover (TC = 30-50%) in discontinuous (10% < P < 90%) permafrost regions (Fig. S4). Therefore, we focused on contrasting trends of net CO_2_ uptake between short-vegetated permafrost (TC < 50%) and forest dominated non-permafrost (TC > 50%) regions. The ACI ensemble showed that net CO_2_ uptake in permafrost regions has increased at a significantly faster rate (0.58 ± 0.086 gC m^−2^ yr^−2^) than in non-permafrost regions (0.13 ± 0.11 gC m^−2^ yr^−2^) from 1980 to 2017 (Fig. S4e). This has caused permafrost regions to switch from being CO_2_ neutral from 1980 to 2000 (3.16 ± 6.51 gC m^−2^yr^−1^) to a CO_2_ sink after 2000 (15.01 ± 6.14 gC m^−2^yr^−1^).

We rule out several factors that potentially confound the observed increase in net CO_2_ uptake in short-vegetated permafrost regions by: (1) considering the uncertainties of ACI estimates resulting from the variance across individual ACIs, partitioning of fluxes between regions, and time-dependent differences in ACI spread (supplementary text); (2) assessing individual ACIs, where all of the inversions showed increasing net CO_2_ uptake in the permafrost region (Fig. S5); (3) conducting a sensitivity test on the time period considered (Fig. S6); and (4) showing that both large-scale patterns and site-level EC measurements had an increasing net CO_2_ uptake over permafrost regions, despite occurring at different rates due to scale mismatch between ACIs and EC footprints (Fig. S7); (5) assessing spatial and seasonal consistency of trends from individual ACIs (Fig. S8); and (6) verifying that the addition of more ACIs after 2000 did not significantly alter the trends (Fig. S9 and S6). Therefore, despite generally large uncertainties among ACIs, our results are robust against outliers and in agreement with independent observations from EC data. Therefore, based on the most current atmospheric inversion estimates, the carbon sink strength of shrub and graminoid-dominated permafrost regions has been increasing significantly faster than tree-dominated non-permafrost regions in the NHL.

### Mechanisms underlying different net CO_2_ uptake trends along vegetation gradients

**Fig. 2:**
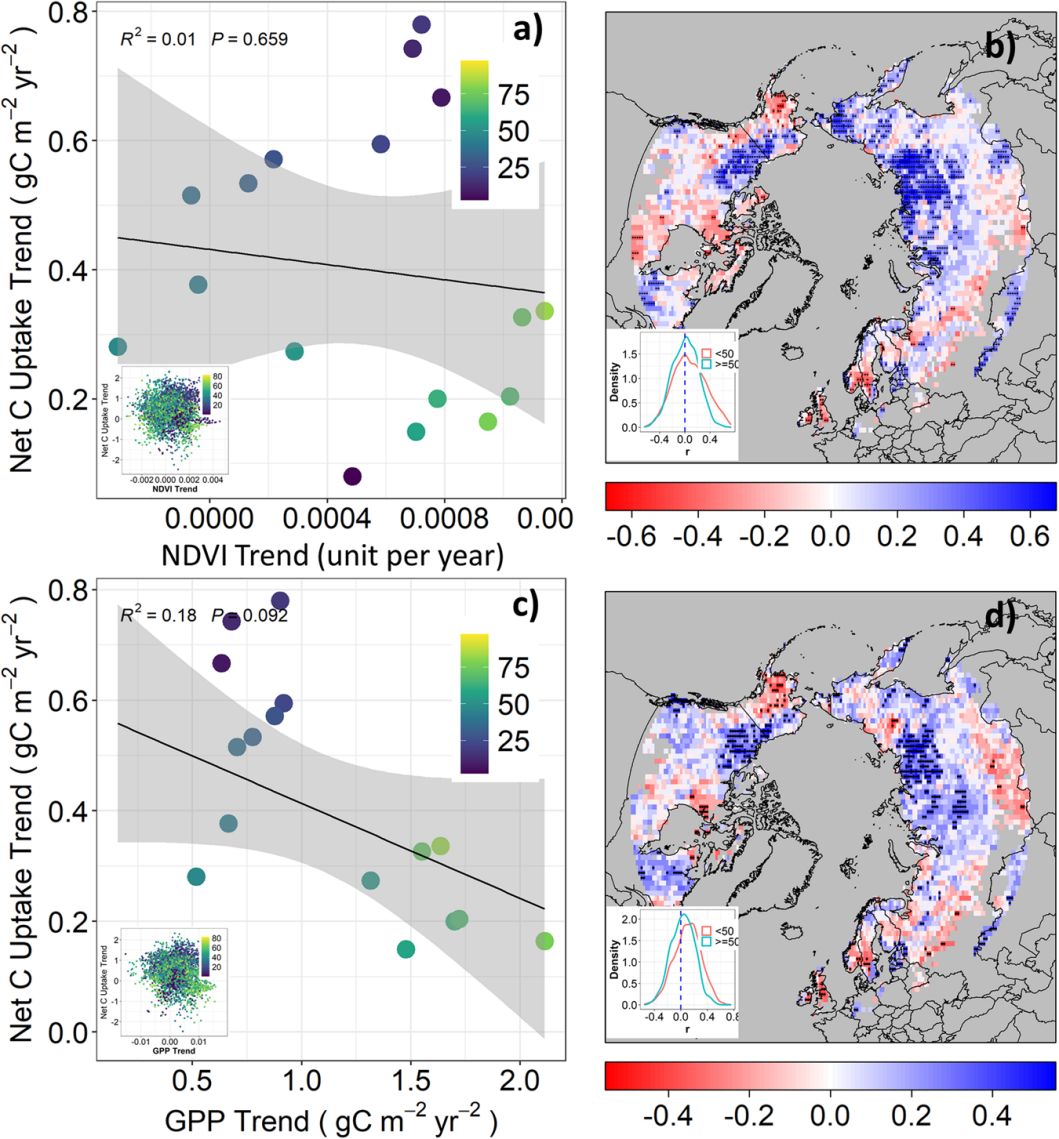
Weak negative correlations between trends of productivity and net CO_2_ uptake suggesting productivity alone cannot explain the different trends of net CO_2_ uptake along the tree cover gradient. **a**, **c** show correlation between trends of GIMMS NDVI or LUE GPP and net CO_2_ uptake trends along the 5% tree cover interval gradient (blue to green), respectively. Insets show the correlation at pixel level. **b** and **d** are spatial patterns of correlation (r) between GIMMS NDVI or LUE GPP and net CO_2_ uptake, respectively. Points indicate trends are significant at 0.05 level. Insets show the distribution of r between regions with TC > = 50% and TC < 50%.

Plant photosynthesis is generally thermally-limited and warming-induced relaxation of cold temperature constraints on ecosystem productivity is a key driver for terrestrial CO_2_ sink dynamics^21^ and enhanced CO_2_ uptake in the NHL^22^. We therefore tested the first hypothesis (H1) that different trends in net CO_2_ uptake along the tree cover gradient were due to differences in vegetation productivity. Physiologically, the larger temperature constraints and amplified rate of warming further north^23^ may result in a greater temperature response of vegetation productivity and therefore explain the higher trend of net CO_2_ uptake in tundra permafrost regions. Long-term satellite observations of NDVI and GPP derived from light use efficiency models confirm a generally positive trend in annual vegetation productivity along the tree cover gradient (Fig. 2a, c, x-axis), consistent with documented NHL greening trends^8^. We also find a widespread positive correlation between productivity and net CO_2_ uptake (Fig. 2b, d), suggesting that increasing net CO_2_ uptake is driven by warming-induced increases in productivity. However, the trends of productivity and net CO_2_ uptake showed a non-significant (*p* > 0.05) correlation along the tree cover gradient (Fig. 2a, c). In fact, trends in annual productivity increased with tree cover (Fig. S10), and were therefore inconsistent with the trends of net CO_2_ uptake shown by ACIs. There are also no apparent trends in annual productivity increase and net CO_2_ uptake along the NHL permafrost and temperature gradients (results not shown). Therefore, warming-induced relaxation of temperature constraints appears to be an important driver of increased productivity and net CO_2_ uptake across NHL ecosystems, but cannot explain the different trends of net CO_2_ uptake seen by ACIs along regional tree cover, temperature, and permafrost gradients.

**Fig. 3:**
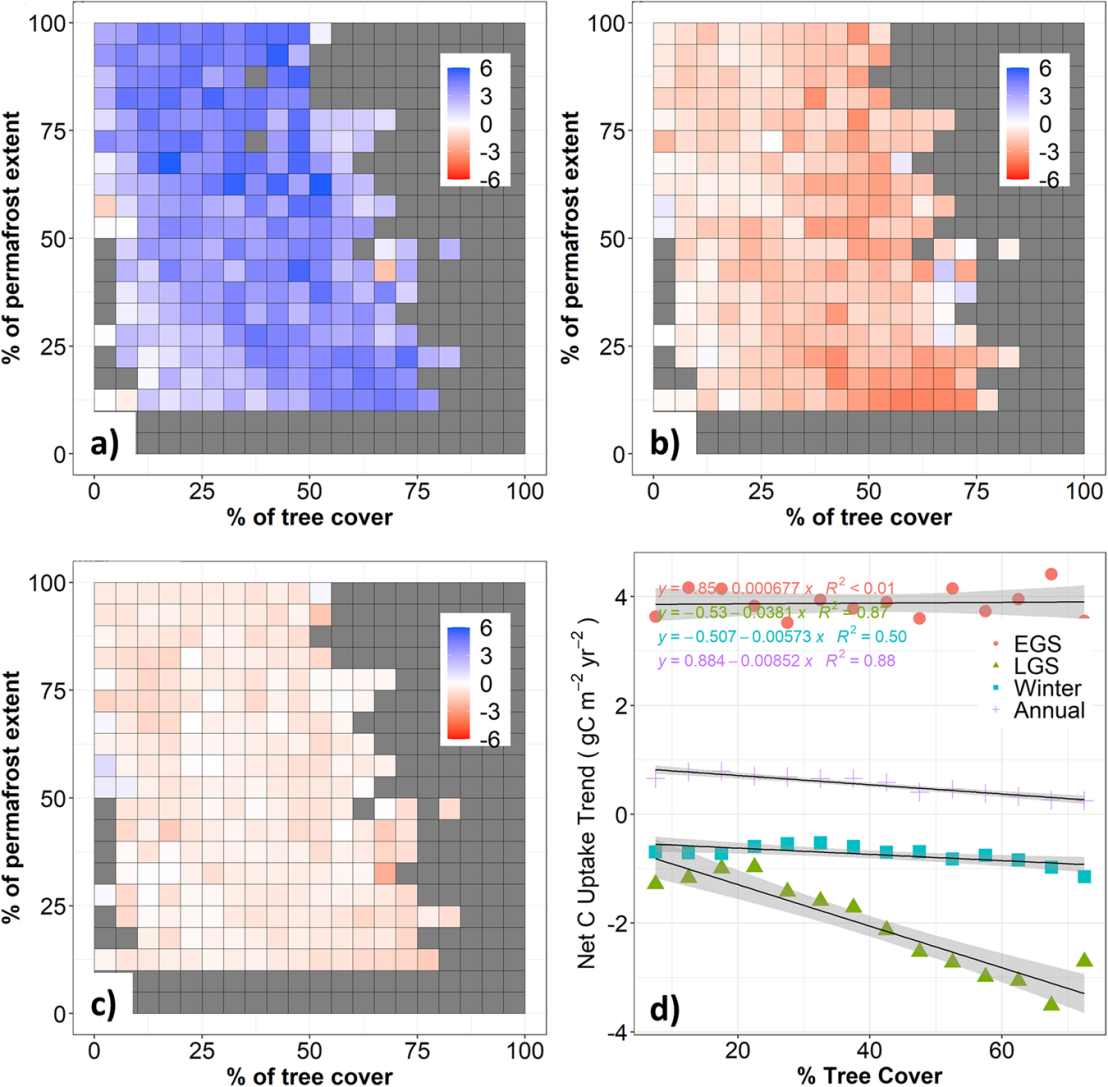
Seasonal compensation explains the different trends of net CO_2_ uptake along tree cover and permafrost gradients in the NHL. **a**–**c** show increasing net CO_2_ uptake in the early-growing season (**a**; EGS: May-Aug) and increasing net CO_2_ release in the late-growing season (**b**; LGS: Sep-Oct) and winter (**c**; Win: Nov-Apr). **d** shows the variation in net CO_2_ uptake seasonal trends along the 5% tree cover interval gradient in the NHL. Pixels with grey color represent environmental conditions that do not exist.

We then tested the second hypothesis (H2) that seasonal compensation of net CO_2_ uptake is the main mechanism explaining the different trends of annual net CO_2_ uptake along the tree cover gradients. Across the NHL, accelerating net CO_2_ uptake in the early growing season (EGS; May- August) was partially compensated by an accelerating net CO_2_ release in the late-growing season (LGS; Sep -Oct), thus resulting in weaker trends in annual net CO_2_ uptake (Fig. 3, Fig S11). While the trends of net CO_2_ uptake increase in the EGS are similar regardless of tree cover, the trends of net CO_2_ release in the LGS increased significantly with greater tree cover further south (Fig. 3d), and thus resulted in a much slower rate of net annual CO_2_ uptake and a larger magnitude of seasonal compensation of net CO_2_ uptake with greater tree cover (Fig. 1d).

Aggregating over larger regions allows us to more confidently quantify the seasonal compensation of net CO_2_ uptake. While the trends of net CO_2_ uptake increase were not significantly different between NHL permafrost and non-permafrost regions, increased emissions in the LGS are significantly lower in permafrost (−1.41 ± 0.15 gC m^−2^ yr^−2^) than in non-permafrost (−2.37 ± 0.19 gC m^−2^ yr^−2^) regions (Fig. S4e), therefore resulting in a much higher annual rate of net CO_2_ uptake increase in permafrost. Consequently, short-vegetated permafrost regions showed a positive sensitivity of annual net CO_2_ uptake to mean annual temperature (101 ± 32 TgC yr^−1^ K^−1^, *p* < 0.001) whereas tree-dominated non-permafrost regions had a non-significant negative sensitivity (−14 ± 18 TgC yr^−1 ^K^−1^, *p* = 0.46) in the NHL (Fig. S12). These results indicate that net CO_2_ uptake continued to increase in response to warming in permafrost regions, but the positive temperature response has stalled in non-permafrost regions across the NHL due to enhancement in late growing season respiration.

**Fig. 4:**
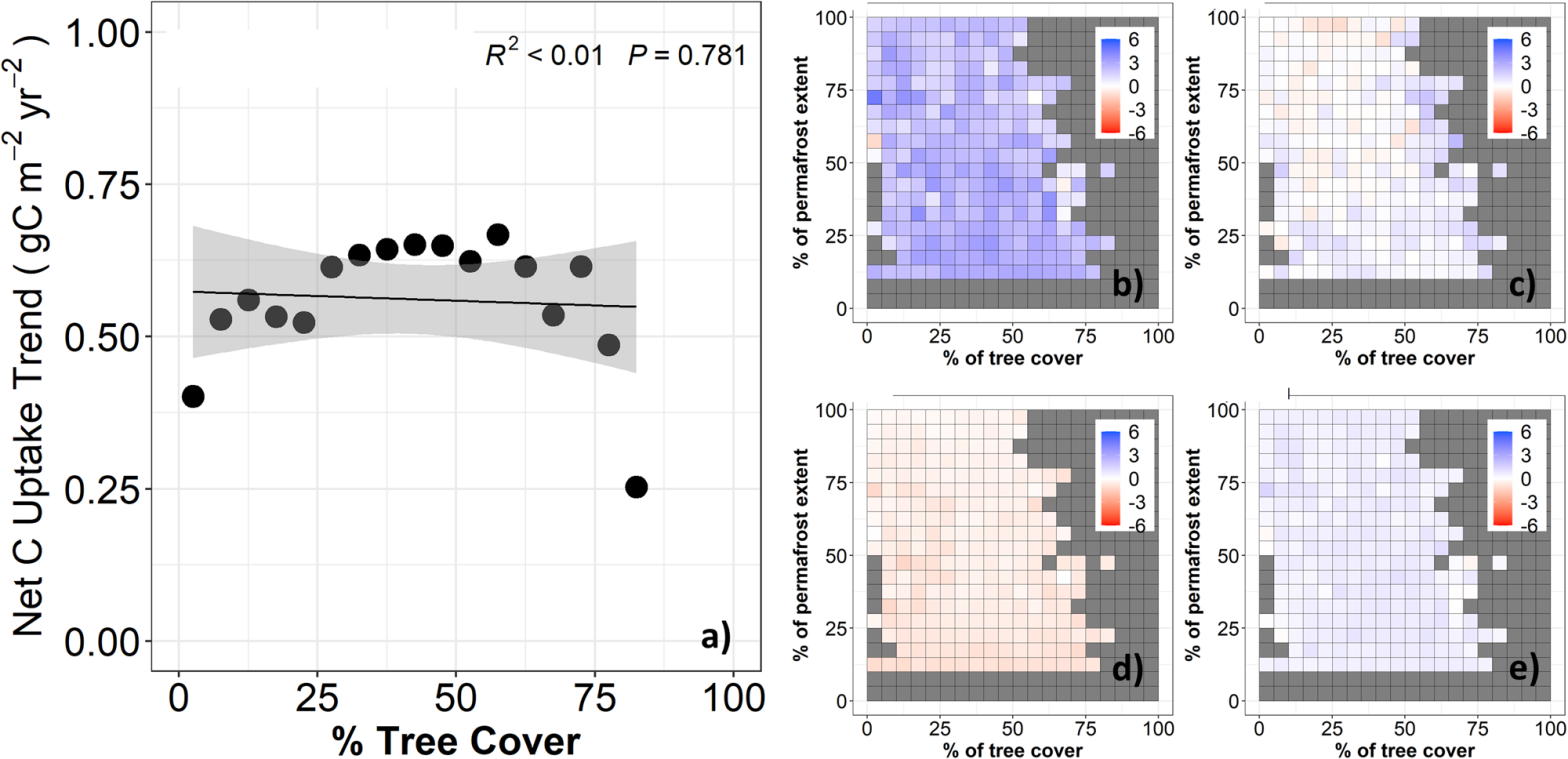
DGVMs cannot capture the decreasing trend of net CO_2_ uptake along the NHL tree cover gradient, mainly because the DGVMs failed to simulate increased CO_2_ emission in the LGS. **a** Correlation between trends of net CO_2_ uptake with tree cover using the TRENDY ensemble. **b**–**e** show net CO_2_ uptake in the early-growing season (EGS: May-Aug), late-growing season (LGS: Sep-Oct), winter (Win: Nov-Apr), and annually along the tree cover and permafrost gradient in the NHL, respectively. Pixels with grey color represent environmental conditions that do not exist.

DGVMs have been widely used to explore climate-vegetation-carbon feedbacks and provide recommendations for policy makers^18^. Although net biome production (NBP) simulated by DGVMs showed that annual net CO_2_ uptake is increasing, there are no apparent model trends of net CO_2_ uptake along the tree cover gradients across the NHL (Fig. 4). This is mainly because DGVMs failed to simulate increased CO_2_ release in LGS, thus underestimating the seasonal compensation of net CO_2_ uptake compared with the observationally-constrained data (Fig. 4), which hinders their ability to capture the changing seasonal atmospheric CO_2_ amplitude^1^ and its attribution^2^. We try to identify the reasons for this DGVM underestimation below.

### Climate and environmental controls on seasonal net CO_2_ uptake compensation

**Fig. 5:**
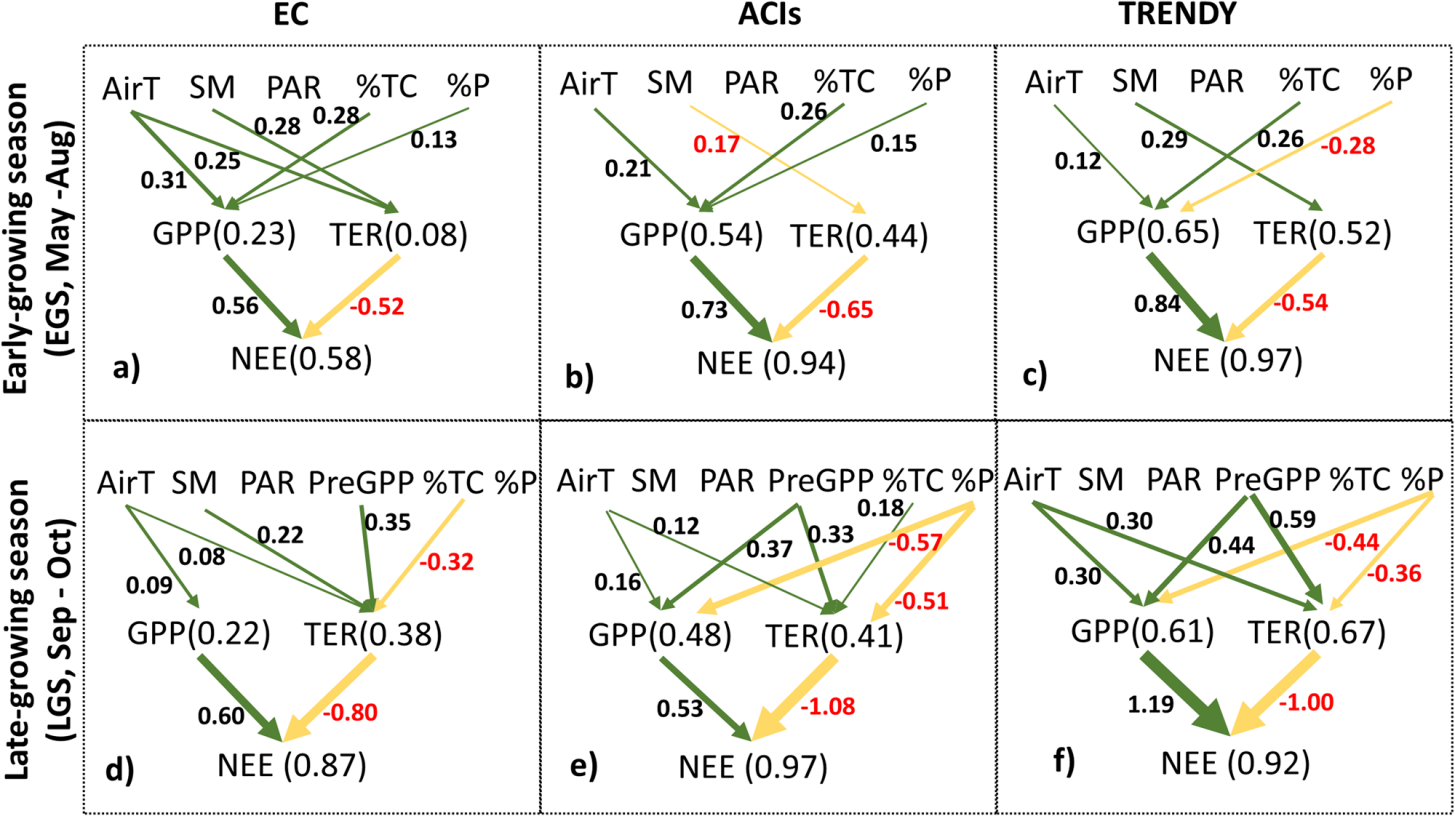
Climate and environmental controls on net CO_2_ uptake vary among seasons, and DGVMs need to better capture the respiration processes during the late-growing season. Structural equation models (SEMs) of the relative influence of component fluxes (i.e., gross primary productivity (GPP) and total ecosystem respiration (TER)) on net CO_2_ uptake (i.e., net ecosystem exchange: NEE) and climatic and environmental controls including air temperature (AirT), soil moisture (SM), photosynthetically active radiation (PAR), preseason GPP (PreGPP), percent tree cover (%TC), and percent of permafrost extent (%P) on CO_2_ fluxes in the NHL. Only significant paths are shown (*p* < 0.05) with the standardized regression coefficient, and thicker (thinner) lines indicating stronger (weaker) relationships. Numbers in brackets after CO_2_ fluxes (i.e., GPP, TER, NEE) indicate the total endogenous (dependent) variation explained by all exogenous (independent) variables in the regression model. Positive (green arrows) and negative (yellow arrows) coefficients indicate respective positive and negative relationships. In the figures, **a**, **b**, **c** are EC, ACI, and TRENDY results, respectively, for the early growing season CO_2_ cycle; whereas **d**, **e**, **f** are EC, ACI, and TRENDY results, respectively, for the late growing season CO_2_ cycle.

Our analysis identified the seasonal compensation of net CO_2_ uptake as key to understanding the climate-carbon feedback in the NHL. We then used structural equation models (SEMs) to gain insights into the underlying carbon cycle processes and environmental controls contributing to NHL seasonal carbon cycle dynamics. SEM is a multivariate, hypothesis-driven method that is based on a structural model representing a hypothesis about the causal relations among interacting variables. For the EGS (Fig. 5a–c), we considered the relative influences of air temperature (Air T), photosynthetically active radiation (PAR), soil moisture content (SM), percent of tree cover (%TC), and percent of permafrost extent (%P) on gross CO_2_ fluxes (i.e., productivity and respiration). All data (EC, ACIs, and TRENDY simulations) showed that net CO_2_ uptake was more strongly regulated by ecosystem productivity, which was itself primarily controlled by spring Air T and %TC. This was consistent with previous analyses indicating that temperature-controlled photosynthetic activity and increase in woody vegetation cover were among the major drivers of productivity and net CO_2_ uptake in the EGS^4,24^.

In the LGS (Fig. 5d, f), we tested whether net CO_2_ release was controlled by moisture stress-induced productivity declines^11^ or increased respiration^25^, either from warming-enhanced respiration rate or increased labile carbon carried over from enhanced productivity early in the growing season (PreGPP)^10,26^. Observationally-constrained estimates of net CO_2_ fluxes (EC and ACIs) showed that respiration had a stronger control on net CO_2_ uptake in the LGS. In contrast, TRENDY simulations showed that productivity continued to have stronger control on net CO_2_ uptake in the LGS. All data showed that EGS productivity had the strongest influence on LGS respiration, followed by temperature. %P had a negative influence on LGS respiration, suggesting warmer conditions result in higher LGS respiration. Based on the observationally-constrained data, net CO_2_ uptake in the LGS was primarily regulated by respiration, which was mainly controlled by increased labile carbon from enhanced early season productivity and temperature. Therefore, larger increases in productivity as tree cover increases (Fig. S10), together with warming, explain the differences in late growing season respiration along the tree cover gradient (Fig. S13). However, given the strong influence of temperature on EGS productivity (Fig. 5a–c), this indirect effect of warming (via increased input of labile carbon) may also have substantial controls on the increased respiratory CO_2_ loss during LGS. Additional analysis also confirmed that warming has both stronger direct and legacy effects on LGS net CO_2_ release in the NHL non-permafrost forested regions (Fig. S15), and therefore contributes to a larger seasonal amplitude of net CO_2_ fluxes, smaller annual net CO_2_ uptake sensitivity to spring temperature, and slower rate of increase in net CO_2_ uptake compared to short-vegetated permafrost regions.

## Discussion

Recent observational^27^, experimental^28,29^, and model simulation studies^30^ have raised concerns that warming-induced acceleration of soil decomposition in permafrost regions may result in a regime shift of arctic ecosystems from a net sink to a net source of CO_2_ to the atmosphere^31,32^. In contrast, our analysis suggests that net CO_2_ uptake has insofar increased in NHL permafrost regions at a faster rate than in tree dominated non-permafrost regions and that most of these permafrost tundra regions have actually shifted from CO_2_ sources to sinks, thus becoming important contributors to the increased CO_2_ sink in the northern hemisphere over the past few decades^3,33^. Such increases in net CO_2_ uptake are primarily driven by warming-enhanced early growing season productivity and increases in shrub/tree vegetation cover^34–37^. Due to the coarse resolution of ACI data, we cannot identify particular ecosystem types contributing to the enhanced net CO_2_ uptake, but our results suggest that ecosystems underlaid by intermediate levels of permafrost extent may be a particularly strong contributor to enhanced net C uptake (Fig. 1f) partially due to higher rate of woody cover expansion (Fig. S14). In addition, our analysis indicates northeast Eurasia as a particularly strong contributor to the net CO_2_ uptake in the NHL (Fig. 1e), possibly because of higher rates of climate warming^38^, less water limitation on ecosystem productivity in continuous permafrost areas, regional wetting from enhanced atmospheric moisture transport^39,40^, higher light use efficiency of deciduous larch forests^41^, regional expansion of tree and shrub cover^42,43^, sustained nitrogen deposition^44^, and less severe fire disturbance compared to arctic-boreal North America^45,46^. However, as tree migration will likely lag climate change, warming-induced species reassembly and permafrost degradation will significantly alter the above- vs belowground carbon cycle and its interaction^47^, especially in climate-sensitive permafrost regions with intermediate tree cover^48^.

On the other hand, the net CO_2_ uptake in NHL forest dominated regions, such as mixed and evergreen needleleaf boreal forest (Fig. S1), has been increasing at a slower rate, primarily due to enhanced late-season CO_2_ release as a result of the warming-induced increase in respiration (Figs. 3 and S13) and emerging moisture limitations on productivity^11^. Under future warmer and drier conditions, enhanced late-season respiratory CO_2_ losses, together with an intensifying fire regime, and moisture stress-induced decreases in productivity, may fully offset or even exceed the net CO_2_ gain from photosynthesis, switching the NHL forested region to a predominant net CO_2_ source. Our analysis showed that both productivity and respiration are important drivers of the seasonal amplitude of net ecosystem CO_2_ uptake in the NHL, but with varied magnitudes and mechanisms across gradients of tree cover, climate, and permafrost. A warming-induced increase of early growing season productivity is the major driver for enhanced net CO_2_ uptake in the NHL (Fig. 3), and this mechanism has been supported by model-based analysis^2^ and advancement of the spring zero-crossing date^49^. On the other hand, increasing respiratory CO_2_ emission in late growing season of forested ecosystems compensate the CO_2_ uptake in early growing season (Fig. 3), and this mechanism is supported by delay of the fall zero-crossing date^25,49^ and airborne and field observations of fall CO_2_ fluxes^9,10^. Moreover, increasing CO_2_ respiration during the late growing season with greater tree cover produces a larger seasonal compensation of net CO_2_ uptake, indicating boreal forest regions may be a stronger driver for the enhanced atmospheric CO_2_ amplitude than arctic tundra. Therefore, our findings help to identify key regions, processes, and seasons to explain the changes in atmospheric CO_2_ seasonal amplitude and reconcile previous studies on whether the increasing NHL atmospheric CO_2_ seasonal amplitude is due to enhanced productivity^2^ or warming-induced early winter respiration^10^.

Our results underscore the importance of the late-growing season CO_2_ cycle in regulating seasonal and annual CO_2_ dynamics in the NHL. The late-growing season net CO_2_ balance has shown contrasting responses to warming^25,49–51^, due to complex interactions among temperature, moisture availability, and radiation on ecosystem productivity^11,52,53^ and respiration^10,25,26^. Our results indicate that the late growing season net CO_2_ balance was strongly influenced by respiration, which is sensitive to change of early season productivity and vegetation change induced by warming (Fig. 5) and may increasingly dominate the future carbon balance of NHL ecosystems. However, the current DGVMs underestimated the late-growing season respiration control on net CO_2_ loss and therefore failed to capture the full magnitude of seasonal CO_2_ dynamics in the high latitudes (Figs. 4 and 5). This helps explain why DGVM-based assessments tend to underestimate the seasonal atmosphere CO_2_ amplitude in the NHL^1,54,55^ and attribute long-term changes largely to boreal productivity^2^. A satellite-driven carbon-flux model (SMAP L4C) that accounted for both atmosphere and soil moisture related controls on net CO_2_ exchange showed similar behavior as the DGVMs (Fig. S15). To better simulate late-growing season respiration and its impact on the net CO_2_ balance, these models may need to include additional processes, such as direct and lagged effects among temperature, moisture availability, and productivity^11^, and the resultant influences on decomposition and turnover of soil organic carbon^56,57^.

In summary, there is currently no evidence that permafrost-dominated regions are, as a whole, a net source of CO_2_ to the atmosphere. On the contrary, net CO_2_ uptake over tundra dominated permafrost regions has been increasing at a faster rate than tree dominated non-permafrost regions in the NHL since the 1980s. As a result, the NHL permafrost region is becoming a strong contributor to the northern terrestrial carbon sink. The faster rate of CO_2_ sink increase in NHL regions is mainly due to enhanced CO_2_ uptake in the early growing season, which is outpacing carbon emissions in the late growing season. On the other hand, the larger seasonal compensation in net CO_2_ uptake as tree cover increases is an important driver of the increasing seasonal atmospheric CO_2_ amplitude in the NHL. Current DGVMs need to better simulate respiration processes in response to changing landscape conditions in order to capture the full magnitude of seasonal CO_2_ dynamics and generate more accurate predictions of climate-carbon feedbacks in northern high latitudes. Although we do not see any evidence yet of positive carbon-climate feedbacks in high latitude ecosystems, thresholds in CO_2_ dynamics may occur as these ecosystems transition in response to the changing state of permafrost.

## Methods

We focused on the Northern High Latitudes (NHL, latitude > 50°N, excluding Greenland) due to their importance for carbon (CO_2_-C, the same hereafter)-climate feedbacks in the Earth system. To minimize the potential human influence on the CO_2_ cycle, we excluded areas under agricultural management (croplands, cropland/natural vegetation mosaic, and urban types), and considered only pixels of natural vegetation defined from the MODIS MCD12Q1 (v006) based IGBP land cover classification. Our main focus was the NHL permafrost region because permafrost plays a critical role in the ecology, environment, and society in the NHL. Permafrost, or permanently frozen ground, is defined as ground (soil, sediment, or rock) that remains at or below 0 °C for at least two consecutive years. The occurrence of permafrost is primarily controlled by temperature and has a strong effect on hydrology, soils, and vegetation composition and structure. Based on the categorical permafrost map from the International Permafrost Association^58^, the permafrost region (excluding permanent snow/ice and barren land), including sporadic (10–50%), discontinuous (50–90%), and continuous (>90%) permafrost, encompasses about 15.7 × 10^6^ km^2^, accounts for 57% of the NHL study dominion, and is dominated by tundra (shrubland and grass) and deciduous needleleaf (i.e., larch) forest that is regionally abundant in Siberia. The NHL non-permafrost region covers about 11.9 × 10^6^ km^2^ and is dominated by mixed and evergreen needleleaf boreal forests (Fig. S1).

### Atmospheric CO_2_ inversions (ACIs)

ACIs provide regionally-integrated estimates of surface-to-atmosphere net ecosystem CO_2_ exchange (NEE_ACI_) fluxes by utilizing atmospheric CO_2_ concentration measurements and atmospheric transport models^59^. ACIs differ from each other mainly in their underlying atmospheric observations, transport models, spatial and temporal flux resolutions, land surface models used to predict prior fluxes, observation uncertainty and prior error assignment, and inversion methods. We used an ensemble mean of six different ACI products, each providing monthly gridded NEE_ACI_ at 1-degree spatial resolution, including Carbon‐Tracker 2019B (2000-2019, CT2019)^60^, Carbon‐Tracker Europe 2020 (2000–2019, CTE2020)^61^, Copernicus Atmosphere Monitoring Service (1979–2019, CAMS)^62^, Jena CarboScope (versions s76_v4.2 1976–2017, and s85_v4.2 1985-2017)^63,64^, and JAMSTEC (1996–2017)^65^. The monthly gridded ensemble mean NEE_ACI_ at 1-degree spatial resolution was calculated using the available ACIs from 1980-2017. Monthly ACI ensemble mean NEE_ACI_ data were summed to seasonal and annual values, and used to calculate the spatial and temporal trends of net CO_2_ uptake, and to investigate its relationship to climate and environmental controls.

### Productivity dataset

Direct observations of vegetation productivity do not exist at a circumpolar scale. We therefore used two long-term gridded satellite-based estimates of vegetation productivity, including gross primary production (GPP) derived using a light use efficiency (LUE) approach (LUE GPP, 1982–1985)^21,66^ and satellite observations of Normalized Difference Vegetation Index (NDVI) from the Global Inventory Modeling and Mapping Studies (GIMMS NDVI, 1982–1985)^67^. LUE GPP (monthly, 0.5° spatial resolution, 1982–2015) is calculated from satellite observations of NDVI from the Advanced Very High-Resolution Radiometer (AVHRR; 1982 to 2015) combined with meteorological data, using the MOD17 LUE approach. LUE GPP has been extensively validated with a global array of eddy-flux tower sites^68–70^ and tends to provide better estimates in ecosystems with greater seasonal variability at high latitudes. Following^66,71^, we used the ensemble mean of GPP estimates from three of the most commonly used meteorological data sets: National Centers for Environmental Prediction/National Center for Atmospheric Research (NCEP/NCAR) reanalysis; NASA Global Modeling and Assimilation Office (GMAO) Modern-Era Retrospective analysis for Research and Applications, Version 2 (MERRA-2); and European Center for Medium-Range Weather Forecasting (ECMWF). GIMMS NDVI (bimonthly, 1/12 spatial resolution, 1982–2015) provides the longest satellite observations of vegetation “greenness”, and is widely used in studies of phenology, productivity, biomass, and disturbance monitoring as it has proven to be an effective surrogate of vegetation photosynthetic activity^72^.

The gridded GPP data were resampled to 1-degree resolution at monthly time scales, to be consistent with NEE_ACI_, and used to test (H1) whether greater temperature sensitivity of vegetation productivity explains the different trends in net CO_2_ uptake across the NHL. LUE GPP was also used to calculate monthly total ecosystem respiration (TER) as the difference between GPP and NEE_ACI_ (i.e., TER_residual_ =  GPP– NEE_ACI_) from 1982-2015, as global observations of respiration do not exist. The NEE_ACI_, GPP and TER_residual_ were used as observation-constrained top-down CO_2_ fluxes to investigate mechanisms underlying the seasonal CO_2_ dynamics in the structural equation modeling and additional decision tree-based analysis.

### Eddy Covariance (EC) measurements of bottom-up CO_2_ fluxes

A total of 48 sites with at least three years of data representing the major NHL ecosystems were obtained from the FLUXNET2015 database (Table S1 and Fig. S1). EC measurements provide direct observations of net ecosystem CO_2_ exchange (NEE) and estimate the GPP and TER flux components of NEE using other climate variables. Daily GPP and TER were estimated as the mean value from both the nighttime partitioning method^73^ and the light response curve method^74^. More details on the flux partitioning and gap-filling methods used are provided by^75^. Daily fluxes were summed into seasonal and annual values and used to compare with trends from ACIs (Fig. S7), to estimate the climate and environmental controls on the CO_2_ cycle in the pathway analysis (Fig. 5), and to calculate the net CO_2_ uptake sensitivity to spring temperature (Fig. S14).

### Ensemble of dynamic global vegetation models (TRENDY simulations)

The TRENDY intercomparison project compiles simulations from state-of-the-art dynamic global vegetation models (DGVMs) to evaluate terrestrial energy, water, and net CO_2_ exchanges^76^. The DGVMs provide a bottom-up approach to evaluate terrestrial CO_2_ fluxes (e.g., net biome production [NBP]) and allow deeper insight into the mechanisms driving changes in carbon stocks and fluxes. We used monthly NBP, GPP, and TER (autotrophic + heterotrophic respiration; Ra + Rh) from ten TRENDY v7 DGVMs^76^, including CABLE-POP, CLM5.0, OCN, ORCHIDEE, ORCHIDEE-CNP, VISIT, DLEM, LPJ, LPJ-GUESS, and LPX. We analyzed the “S3” simulations that include time-varying atmospheric CO_2_ concentrations, climate, and land use. All simulations were based on climate forcing from the CRU-NCEPv4 climate variables at 6-hour resolution. CO_2_ flux outputs were summarized monthly at 1-degree spatial resolution from 1980 to 2017. Monthly ensemble mean NBP, GPP, and TER were summed to seasonal and annual values, and then used to compare with observation-constrained ACI top-down CO_2_ fluxes (Figs. 4 and 5).

### Satellite data-driven carbon flux estimates (SMAP L4C)

We also used a much finer spatio-temporal simulation of carbon fluxes from the NASA Soil Moisture Active Passive (SMAP) mission Level 4 Carbon product (L4C) to quantify the temperature and moisture sensitivity of NHL CO_2_ exchange^77^. The SMAP L4C provides global operational daily estimates of NEE and component CO_2_ fluxes for GPP and TER at 9 km resolution since 2015; whereas, an offline version of the L4C model provides a similar Nature Run (NR) carbon flux record over a longer period (2000-present), but without the influence of SMAP observational inputs. The L4C model has been calibrated against FLUXNET tower CO_2_ flux measurements and shows favorable performance and accuracy in high latitude regions^4,77^. In this analysis, daily gridded CO_2_ fluxes at 9-km resolution from the L4C NR record were summed to seasonal and annual values, and used to calculate the sensitivity of net C uptake in response to spring temperature (Fig. S14).

CO_2_ fluxes in this analysis are defined with respect to the biosphere so that a positive value indicates the biosphere is a net sink of CO_2_ absorbed from the atmosphere. The different data products described above use different terminology (e.g., NEE, NBP) with slightly different meanings; however, they all provide estimates of net land-atmosphere CO_2_ exchange^78^.

### Climate, tree cover, permafrost, and soil moisture data

Monthly gridded air temperatures at 0.5-degree spatial resolution from 1980 to 2017 were obtained from the Climate Research Unit (CRU TS v4.02) at the University of East Anglia^79^. Air temperature was summarized at seasonal and annual scales to calculate temperature sensitivities of net CO_2_ uptake and to investigate the mechanism underlying the seasonal CO_2_ dynamics.

Percent tree cover (%TC) at 0.05-degree spatial resolution was averaged over a 35-year (1982-2016) period using annual %TC layers derived from the Advanced Very High-Resolution Radiometer (AVHRR) (Fig. 1a)^42^. %TC was binned using 5% TC intervals to assess its relation to net CO_2_ uptake, or aggregated at a regional scale (e.g., TC > 50% or TC < 50%) to contrast variation of net CO_2_ uptake or used to explore the mechanism underlying the seasonal CO_2_ dynamics.

We used two permafrost maps, including a continuous permafrost extent map from European Space Agency’s (ESA) Climate Change Initiative (CCI) Permafrost project (Permafrost_CCI, 1-km spatial resolution)^80^ and a categorical permafrost zone map from IPA (International Permafrost Association, Permafrost_IPA, vector data)^58^. The Permafrost_CCI product was derived from a thermal model driven and constrained by satellite observations of land surface temperature. The percent of permafrost extent (%P) for each year was calculated as the yearly fraction of permafrost-underlain (ground temperatures at 2 m depth < 0) and permafrost-free area within a pixel. The %P was averaged over 1997–2017 and aggregated to 0.05-degree spatial resolution (Fig. 1b). The %P metric corresponds well with Permafrost_IPA permafrost zones, which distinguish isolated (0–10%), sporadic (10–50%), discontinuous (50–90%) and continuous permafrost (90–100%). In this analysis, the %P map was binned at 5% intervals to show its relation to net CO_2_ uptake or used to explore the mechanism underlying the seasonal CO_2_ dynamics. The permafrost map was also aggregated into continuous permafrost (ConP, *P* > 90%), discontinuous permafrost (DisconP, 10% < *P* < 90%), and non-permafrost (NoP, *P* < 10%) zones for regional analysis.

We used the ESA CCI soil moisture product (SM, v4.5) produced from combined satellite active and passive microwave remote sensing observations^81^ at daily, 0.25-degree spatial resolution from 1980 to 2017. The ESA CCI SM product was developed using data derived from C-band scatterometers suitable for SM retrieval, such as European Remote Sensing Satellites (ERS-½) and METOP, as well as the use of data from multi-frequency microwave radiometers such as the Scanning Multichannel Microwave Radiometer (SMMR), Special Sensor Microwave Imager (SSM/I), Microwave Imager (TMI), Advanced Microwave Scanning Radiometer (AMSR-E), and Windsat^81^.

The ESA CCI SM product characterizes surface (0–5 cm depth) SM conditions that are highly variable at daily time scales. We first aggregated the daily gridded SM estimates into monthly mean gridded SM to reduce the temporal variability and spatial gaps. Monthly gridded SM estimates were then aggregated into seasonal and annual averages and used to explore their relationships with the land-atmosphere CO_2_ fluxes.

### Net CO_2_ trends along vegetation, climate, and permafrost gradients

We binned %TC and %P into 5% intervals, and annual mean air temperature into 1-degree intervals. The net CO_2_ uptake for the early-growing season (EGS: May–August), late-growing season (LGS: September–October), winter (November–April), and annual periods from 1980 to 2017 was summarized using the ensemble mean of the ACIs (NEE_ACI_) at each binned interval. The seasonal and annual mean net CO_2_ uptake for each interval of %TC, %P, and temperature was then regressed against years using linear regression. The slope of the regression was interpreted as the net CO_2_ uptake trend (gC m^2^ yr^−2^). Finally, trends of net CO_2_ uptake at seasonal and annual scales were plotted against %TC, %P, and air temperature to understand the trend and seasonality of net CO_2_ uptake along the vegetation, climate, and permafrost gradient (Figs. 1 and 3).

### Regional analysis

To reduce the pixel-scale uncertainties of net CO_2_ uptake using ACIs data, we also calculated the trends of net CO_2_ uptake at regional scales, which were classified by %TC and %P (Fig. S4). Using %TC, the NHL was divided into low (< 30%), intermediate (30–50%), and high (>50%) tree cover regions. Using %P, the NHL was divided into continuous (ConP, *P* > 90%), discontinuous (DisconP, 10% < *P* < 90%), and non-permafrost (NoP, *P* < 10%) regions. %TC and %P are highly correlated, such that short-vegetated regions (TC < 50%) are primarily overlying permafrost (ConP and DisconP), whereas the tree-dominated regions (TC > 50%) are primarily in non-permafrost (Fig. S4). Spatial and temporal patterns for the net CO_2_ uptake trend were calculated seasonally and annually from 1980 to 2017 using the ensemble mean of the ACIs (NEE_ACI_) over different NHL regions. Seasonal and annual mean net CO_2_ uptake for each region was regressed against years using linear regression. The slope of the regression was interpreted as the net CO_2_ uptake trend (gC m^2^ yr^−2^, Fig. S4).

### Robustness analysis

Since trends of net CO_2_ uptake are not statistically different between low tree cover (< 30% tree cover in ConP regions) and intermediate tree cover (30-50% tree cover in DisconP regions) (Fig. S4), we aggregated these two regions into a short-vegetated (TC < 50%) permafrost region. We contrasted the net CO_2_ uptake between short-vegetated (TC < 50%) permafrost and tree-dominated (TC > 50%) non-permafrost regions. To confirm the net CO_2_ uptake trend between permafrost and non-permafrost regions obtained by the ACIs, we performed a series of additional analyses as follows.

#### Individual ACI analysis

We repeated the trend analysis on permafrost and non-permafrost regions for each individual ACI. All ACIs showed that net CO_2_ uptake is increasing faster in the permafrost region than in the non-permafrost region, although only three ACIs (CT2019B, CTE2020, CAMS) showed significantly faster trends over the available data periods (Fig. S5).

#### Random years and length analysis

We randomly selected starting years and length of years (>= 10 years) for the ensemble mean ACIs, and then repeated the trend analysis. Results consistently showed that net CO_2_ uptake is increasing at a much faster rate in permafrost regions than in non-permafrost regions (Fig. S6).

#### Site-level analysis

We conducted a site-level comparison between EC observations and the ACI ensemble at NHL tower site locations since the 1990s. Both the EC measurements and ACI ensembles confirmed that the net CO_2_ uptake is increasing with decreasing tree cover over and increasing permafrost extent, such that the short-vegetated permafrost region (%TC < 50%) had a higher rate of net CO_2_ uptake increase than the tree-dominated non-permafrost region (%TC > 50%) during the 1990s – 2010s (Fig. S7). This result is also consistent with an independent EC-based analysis showing that forest sties had a slower increasing rate of net CO_2_ uptake than other ecosystem types (e.g., tundra, wetland) in northern permafrost regions^13^. Expectedly, the net CO_2_ uptake trends calculated by the EC observations are much higher than the ACIs because (1) the ACIs represent a much larger spatial footprint than the ecosystem-level EC measurements, and therefore average out the local-scale variability, and (2) some episodic ecosystem processes, such as fire disturbance, were not accounted for by the EC observations.

#### ACI uncertainty analysis

The uncertainties among different ACIs may also affect trend estimates. Uncertainty in ACIs estimates may be due to (i) spread across inversions, (ii) differences among inversions in partitioning of fluxes between permafrost and non-permafrost regions, and (iii) time-dependent differences in inversion spread. Therefore, we used a Generalized Linear Mixed effects Model (GLMM) to estimate trends, considering (i)-(iii) as random effects. The GLMM showed that even after accounting for the uncertainty due to inversion spread, the rate of net CO_2_ uptake in permafrost regions is still significantly faster than non-permafrost regions (see supplementary text for full details).

#### Spatial trend agreement analysis

to assess the spatial consistency of trends of net CO_2_ uptake derived from individual ACIs, we calculated the number of ACIs that showed similar trends (either decreasing or increasing). If the majority of ACIs (i.e., >= 5 of 6 ACIs) showed similar trends, we considered the trend in these areas to be true. We reported the trends within these areas here. Generally, we found more areas in the EGS showing similar trends than the other seasons, reflecting larger uncertainties in understanding the carbon cycle in LGS and winter season. In the EGS, 76% of the non-permafrost region and 77% of the permafrost region showed increasing net CO_2_ uptake. Annually, only 46% of the non-permafrost region and 51% of the permafrost region showed increasing net CO_2_ uptake because of high uncertainties in the LGS and winter (Fig. S8).

#### Time-series analysis

to see if the trend analysis was affected by including more ACIs in the record after year 2000, we compared the trends calculated from the two longest ACIs (i.e., CAMS, and Jena CarboScope (s76)) with trends calculated from the 3 shortest ACIs (i.e., CT2020B, CTE2020, and JAMSTEC). Results showed that permafrost regions still have a significantly higher rate of net CO_2_ uptake using the two longest continuous ACIs since 1980 (CAMS and Jena CarboScope (s76)). The trend of net CO_2_ uptake is significantly higher after 2000, which may elevate trends in the permafrost region since the 1980s (Fig. S9). Therefore, including more ACIs after 2000 may change the magnitude of trends in permafrost regions, but does not alter our finding that net CO_2_ uptake increased faster in permafrost than non-permafrost regions.

### Comparison with Global Carbon Budget 2020 (GCB2020)

The global land CO_2_ sink and trend between the ensemble of ACIs and the Global Carbon Budget 2020 (GCB2020), which was estimated from the multi-model mean of 16 DGVMs^18^, were also compared from 1980 to 2017 (Fig. S2). We note that this comparison is not a fully independent representation of the land sink, as the ACIs and GCB2020 land-sink estimates used similar fossil fuel emissions, ocean fluxes, and land-use change emissions, and some TRENDY model calculations served as a-priori land-sink for individual ACIs.

### Comparison with TRENDY NBP simulations

To see if current state-of-art land surface simulation models reproduce the trend and seasonality of net C uptake relative to the ACIs, the same analysis of net CO_2_ uptake spatial and temporal patterns and trends was applied to the NBP simulations from TRENDY (Fig. 4).

### Trend and seasonality of productivity

To test H1(whether greater temperature sensitivity of vegetation productivity in the NHL explains the different trends in net CO_2_ uptake along the gradient of tree cover), we first calculated the trend and seasonality of productivity using the two productivity products described above, following the same trend analysis procedure used for the ACIs (Fig. S10). The analysis showed that the trend and seasonality are similar using different productivity proxies despite different algorithms, observations, and driving variables among products. Both annual NDVI and GPP data showed positive trends in the NHL (Fig. S10), consistent with greening trends reported from other studies. Furthermore, trends in productivity generally increased with tree cover (Fig. S10), in direct contrast to the decreasing trends of net CO_2_ uptake with increase in tree cover.

To further test H1, we calculated the correlations between the trends in productivity and net CO_2_ uptake along the tree cover gradient at both pixel level and 5% intervals (Fig. 2a, c). We also calculated the correlation between pixel-level time-series net CO_2_ uptake and productivity from 1982 to 2015 (Fig. 2b, d).

### Path analysis to explore the mechanism underlying the seasonal CO_2_ cycles

Structural equation modeling (SEM) is a powerful multivariate technique to evaluate direct and indirect effects between pre-assumed causal relationships within multivariate data^82^. SEM combines two statistical methods, confirmatory factor analysis and path analysis, and aims to find the causal relationship among variables by creating a path diagram. SEM is well suited to test the complex interaction among CO_2_ cycles and controls.

Based on a priori expectations, we constructed one structural equation model (SEM) for each season (EGS and LGS) to test the relative influence of component CO_2_ fluxes (i.e., productivity [GPP] and respiration [TER]) on the net CO_2_ flux (NEE), and the climate and environmental controls on the NEE component CO_2_ fluxes. Our goal was not to precisely predict the spatial and temporal CO_2_ cycle variability, but rather to illuminate and quantify the relative influence of the major climatic and environmental controls on CO_2_ fluxes. We constructed different models to explain EGS and LGS CO_2_ cycles because of the varied climate and environmental controls across seasons over the NHL^4^. For EGS, we tested the relative influences of air temperature (Air T), photosynthetic active radiation (PAR), soil moisture (SM), percent tree cover (%TC), and percent permafrost extent (%P) on vegetation photosynthetic activity and respiration, and the resultant influence on net CO_2_ uptake^4,24^. For LGS, we tested the relative influences of Air T, PAR, SM, %TC, %P, and labile carbon carried over from the early growing season (PreGPP) on vegetation photosynthetic activity and respiration, and the resultant influence on the net CO_2_ uptake results^4,10,26,52^ (Fig. 5).

SEMs were fit using the sem function of the lavaan package in R^83^. The performances of the SEMs were evaluated using a combination of the chi-square statistic (where χ2 ≤ 2 and p > 0.05 indicate a good fitting model), Bentler’s comparative fit index (CFI, where CFI ≈ 1 indicates a good fitting model), and the root mean square error of approximation (RMSEA; where RMSEA ≤ 0.05 and *p* > 0.1 indicate a good fitting model). The standardized regression coefficient can be interpreted as the relative influences of exogenous (independent) variables. The R^2^ indicates the total variation in an endogenous (dependent) variable explained by all exogenous (independent) variables.

### Direct and legacy effects of temperature on seasonal net CO_2_ uptake

Because landscape thawing and snow conditions regulate the onset of vegetation growth and influence the seasonal and annual CO_2_ cycles in the NHL^24,84^, we also analyzed the legacy effects of spring (May–Jun) temperature on seasonal net CO_2_ uptake. We regressed seasonal and annual net CO_2_ uptake from the site-level EC observations, regional-level ACI ensemble, and the TRENDY NBP ensemble against spring (May-June) air temperature. For EC observations, net CO_2_ uptake (i.e., NEE) and air temperature were summarized from site-level measurements. For the ACIs and TRENDY ensemble, net CO_2_ uptake (i.e., NEE_ACI_ and NBP) was summarized as regional means from the ACIs and TRENDY ensemble outputs, and air temperature was summarized as regional means from CRU temperature. The slope of the regression line was interpreted as the spring temperature sensitivity of the CO_2_ cycle. Simple linear regression was used here mainly due to the strong influence of spring temperature on the seasonal and annual CO_2_ cycle in NHL ecosystems^30^. Temperature sensitivity (*γ*: g C m^−2^ day^−1^ K^−1^) is the change in net CO_2_ flux (g C m^−2^ day^−1^) in response to a 1-degree temperature change. The sensitivity of net CO_2_ uptake to warm spring anomalies was calculated for different seasons (EGS, LGS, and annual) and regions (i.e., permafrost and non-permafrost), and the T-test was used to test for the difference in *γ* among different regions, seasons, and datasets. Similarly, direct effects of temperature on net CO_2_ uptake were calculated using the same season data (Fig. S14).

Observationally-constrained estimates (EC and ACIs) showed that the sensitivity of net CO_2_ uptake in the EGS to spring temperature is positive (*γ* > 0) and not statistically different (*p* > 0.05) between permafrost and non-permafrost regions (${\gamma }_{{ACI}}^{{np}}$=0.125 ± 0.020 gC m^−2^ d^−1^ K^−1^; ${\gamma }_{{EC}}^{{np}}$ = 0.052 ± 0.013 gC m^−2^ d^−1^ K^−1^). In contrast, the sensitivity of net CO_2_ uptake in LGS to spring temperature is negative (*γ* < 0) and significantly smaller in magnitude in permafrost regions (${\gamma }_{{ACI}}^{p}$= −0.054 ± 0.0064 gC m^−2^ d^−1^ K^−1^; ${\gamma }_{{EC}}^{p}$=−0.0157 ± 0.0084 gC m^−2^ d^−1^ K^−1^) than non-permafrost regions (${\gamma }_{{ACI}}^{{np}}$= −0.093 ± 0.0070 gC m^−2^ d^−1^ K^−1^; ${\gamma }_{{EC}}^{{np}}$ = −0.061 ± 0.013 gC m^−2^ d^−1^ K^−1^). Net CO_2_ uptake in the LGS is also negatively correlated to LGS temperature in the non-permafrost region (${\gamma }_{{ACI}}^{{np}}$= −0.056 ± 0.0062 gC m^−2^ d^−1^ K^−1^; ${\gamma }_{{EC}}^{{np}}$ = −0.061 ± 0.011 gC m^−2^ d^−1^ K^-^), but showed a varied response in permafrost regions (${\gamma }_{{ACI}}^{p}$= −0.027 ± 0.0046 -gC m^−2^ d^−1^ K^−1^; ${\gamma }_{{EC}}^{{np}}$ = 0.024 ± 0.011 gC m^−2^ d^−1^ K^−1^_,_ Fig. S14). Overall, spring warming has stronger legacy effects on LGS net CO_2_ release in the NHL non-permafrost regions, and therefore contributes to a larger seasonal amplitude of net CO_2_ fluxes, smaller annual net CO_2_ uptake sensitivity to spring temperature, and slower rate of increase in net CO_2_ uptake compared to permafrost regions.

Consistent with regional estimates from ACIs, NBP simulated by DGVMs showed a positive sensitivity of EGS net CO_2_ uptake to spring temperature (${\gamma }_{{TRENDY}}^{{np}}$= 0.044 ± 0.025 gC m^−2^ d^−1^ K^−1^; ${\gamma }_{{TRENDY}}^{p}$ = 0.111 ± 0.0075 gC m^−2^ d^−1^ K^−1^), contributing to an annual net CO_2_ uptake in the NHL. However, simulated LGS NBP showed non-significant (${\gamma }_{{TRENDY}}^{p}$ = 0.0034 ± 0.0036 gC m^−2^ d^−1^ K^−1^) or positive (${\gamma }_{{TRENDY}}^{{np}}$ = 0.0201 ± 0.0061 gC m^−2^ d^−1^ K^−1^) sensitivities to spring warming, failed to capture the seasonal compensation in net CO_2_ uptake in both permafrost and non-permafrost regions, which therefore partially explained the lack of trends in NBP along the NHL tree cover gradient (Fig. 4).

## Supplementary information

Supplementary figures and tables

Peer Review File

## Data Availability

All data analyzed in this study are publicly available. Carbon tracker is obtained from NOAA Earth System Research Laboratory (https://www.esrl.noaa.gov/gmd/ccgg/carbontracker/), Carbon Tracker Europe from Wageningen University (http://www.carbontracker.eu/), Jena CarboScope is from MPG (http://www.bgc-jena.mpg.de/CarboScope/), and CAMS from ECMWF (http://apps.ecmwf.int/datasets/data/cams-ghg-inversions/). SMAP L4C data are from the National Snow and Ice Data Center (https://nsidc.org/). TRENDY simulation is obtained from http://dgvm.ceh.ac.uk/index.html. Climate data are from Climate Research Unit (https://crudata.uea.ac.uk/cru/data/hrg/). EC data is from FLUXNET2015 Dataset (https://fluxnet.org/data/fluxnet2015-dataset/).

## Code availability

All computer codes used in this study are available via GitHub at https://github.com/liuzh811/PermafrostCarbonCycle, and also archived at Zenodo (https://zenodo.org/badge/latestdoi/510523596).

## References

[1] Graven HD (2013). Enhanced seasonal exchange of CO_2_ by northern ecosystems since 1960. Science.

[2] Forkel M (2016). Enhanced seasonal CO_2_ exchange caused by amplified plant productivity in northern ecosystems. Science.

[3] Ciais P (2019). Five decades of northern land carbon uptake revealed by the interhemispheric CO_2_ gradient. Nature.

[4] Liu Z (2020). Increased high-latitude photosynthetic carbon gain offset by respiration carbon loss during an anomalous warm winter to spring transition. Glob. Chang Biol..

[5] Xia JY (2014). Terrestrial carbon cycle affected by non-uniform climate warming. Nat. Geosci..

[6] Helbig M (2022). Warming response of peatland CO_2_ sink is sensitive to seasonality in warming trends. Nat. Clim. Change.

[7] Myers-Smith IH (2020). Complexity revealed in the greening of the Arctic. Nat. Clim. Change.

[8] Piao S (2019). Characteristics, drivers and feedbacks of global greening. Nat. Rev. Earth Environ..

[9] Natali SM (2019). Large loss of CO2 in winter observed across the northern permafrost region. Nat. Clim. Change.

[10] Commane R (2017). Carbon dioxide sources from Alaska driven by increasing early winter respiration from Arctic tundra. Proc. Natl Acad. Sci. USA.

[11] Buermann W (2018). Widespread seasonal compensation effects of spring warming on northern plant productivity. Nature.

[12] Mekonnen ZA (2021). Arctic tundra shrubification: a review of mechanisms and impacts on ecosystem carbon balance. Environ. Res. Lett..

[13] Li Z-L (2021). Changes in net ecosystem exchange of CO2 in Arctic and their relationships with climate change during 2002–2017. Adv. Clim. Change Res..

[14] Welp LR (2016). Increasing summer net CO2 uptake in high northern ecosystems inferred from atmospheric inversions and comparisons to remote-sensing NDVI. Atmos. Chem. Phys..

[15] Schuur EA (2015). Climate change and the permafrost carbon feedback. Nature.

[16] McGuire AD (2018). Dependence of the evolution of carbon dynamics in the northern permafrost region on the trajectory of climate change. Proc. Natl Acad. Sci. USA.

[17] McGuire AD (2012). An assessment of the carbon balance of Arctic tundra: comparisons among observations, process models, and atmospheric inversions. Biogeosciences.

[18] Friedlingstein P (2020). Global Carbon Budget 2020. Earth Syst. Sci. Data.

[19] Tagesson T (2020). Recent divergence in the contributions of tropical and boreal forests to the terrestrial carbon sink. Nat. Ecol. Evol..

[20] Virkkala A-M (2021). Statistical upscaling of ecosystem CO2 fluxes across the terrestrial tundra and boreal domain: regional patterns and uncertainties. Glob. Change Biol..

[21] Running SW (2004). A continuous satellite-derived measure of global terrestrial primary production. Bioscience.

[22] Liu J, Wennberg PO, Parazoo NC, Yin Y, Frankenberg C (2020). Observational constraints on the response of high-latitude northern forests to warming. AGU Adv..

[23] Serreze MC, Barry RG (2011). Processes and impacts of Arctic amplification: a research synthesis. Glob. Planet. Change.

[24] Parazoo NC (2018). Spring photosynthetic onset and net CO2 uptake in Alaska triggered by landscape thawing. Glob. Chang Biol..

[25] Piao S (2008). Net carbon dioxide losses of northern ecosystems in response to autumn warming. Nature.

[26] Jeong SJ (2018). Accelerating rates of Arctic carbon cycling revealed by long-term atmospheric CO_2_ measurements. Sci. Adv..

[27] Belshe EF, Schuur EA, Bolker BM (2013). Tundra ecosystems observed to be CO_2_ sources due to differential amplification of the carbon cycle. Ecol. Lett..

[28] Plaza C (2019). Direct observation of permafrost degradation and rapid soil carbon loss in tundra. Nat. Geosci..

[29] Mauritz M (2017). Nonlinear CO2 flux response to 7 years of experimentally induced permafrost thaw. Glob. Chang Biol..

[30] Turetsky MR (2020). Carbon release through abrupt permafrost thaw. Nat. Geosci..

[31] Schadel C (2016). Potential carbon emissions dominated by carbon dioxide from thawed permafrost soils. Nat. Clim. Change.

[32] Hugelius G (2020). Large stocks of peatland carbon and nitrogen are vulnerable to permafrost thaw. Proc. Natl Acad. Sci. USA.

[33] Ballantyne AP, Alden CB, Miller JB, Tans PP, White JW (2012). Increase in observed net carbon dioxide uptake by land and oceans during the past 50 years. Nature.

[34] Zhu ZC (2016). Greening of the Earth and its drivers. Nat. Clim. Change.

[35] Bjorkman AD (2020). Status and trends in Arctic vegetation: evidence from experimental warming and long-term monitoring. Ambio.

[36] Myers-Smith IH (2019). Eighteen years of ecological monitoring reveals multiple lines of evidence for tundra vegetation change. Ecol. Monogr..

[37] Bjorkman AD (2018). Plant functional trait change across a warming tundra biome. Nature.

[38] Jansen E (2020). Past perspectives on the present era of abrupt Arctic climate change. Nat. Clim. Change.

[39] Pulliainen J (2020). Patterns and trends of Northern Hemisphere snow mass from 1980 to 2018. Nature.

[40] Lian X (2020). Summer soil drying exacerbated by earlier spring greening of northern vegetation. Sci. Adv..

[41] Welp LR, Randerson JT, Liu HP (2007). The sensitivity of carbon fluxes to spring warming and summer drought depends on plant functional type in boreal forest ecosystems. Agric. For. Meteorol..

[42] Song XP (2018). Global land change from 1982 to 2016. Nature.

[43] Frost GV, Epstein HE (2014). Tall shrub and tree expansion in Siberian tundra ecotones since the 1960s. Glob. Chang Biol..

[44] Liu X (2013). Enhanced nitrogen deposition over China. Nature.

[45] Rogers BM, Soja AJ, Goulden ML, Randerson JT (2015). Influence of tree species on continental differences in boreal fires and climate feedbacks. Nat. Geosci..

[46] Liu Z, Ballantyne AP, Cooper LA (2019). Biophysical feedback of global forest fires on surface temperature. Nat. Commun..

[47] Rees WG (2020). Is subarctic forest advance able to keep pace with climate change?. Glob. Change Biol..

[48] Scheffer M, Hirota M, Holmgren M, Van Nes EH, Chapin FS (2012). Thresholds for boreal biome transitions. PNAS.

[49] Barichivich J (2013). Large‐scale variations in the vegetation growing season and annual cycle of atmospheric CO2 at high northern latitudes from 1950 to 2011. Glob. Change Biol..

[50] Keenan TF (2014). Net carbon uptake has increased through warming-induced changes in temperate forest phenology. Nat. Clim. Change.

[51] Ueyama M, Iwata H, Harazono Y (2014). Autumn warming reduces the CO2 sink of a black spruce forest in interior Alaska based on a nine-year eddy covariance measurement. Glob. Chang Biol..

[52] Zhang Y, Parazoo NC, Williams AP, Zhou S, Gentine P (2020). Large and projected strengthening moisture limitation on end-of-season photosynthesis. Proc. Natl Acad. Sci. USA.

[53] Zhang Y, Commane R, Zhou S, Williams AP, Gentine P (2020). Light limitation regulates the response of autumn terrestrial carbon uptake to warming. Nat. Clim. Change.

[54] Thomas RT (2016). Increased light-use efficiency in northern terrestrial ecosystems indicated by CO2 and greening observations. Geophys. Res. Lett..

[55] Peng SS (2015). Benchmarking the seasonal cycle of CO2 fluxes simulated by terrestrial ecosystem models. Glob. Biogeochemical Cycles.

[56] Crowther TW (2019). The global soil community and its influence on biogeochemistry. Science.

[57] Endsley KA, Kimball JS, Reichle RH (2022). Soil Respiration Phenology Improves Modeled Phase of Terrestrial net Ecosystem Exchange in Northern Hemisphere. J. Adv. Modeling Earth Syst..

[58] Brown, J., Ferrians Jr, O., Heginbottom, J. & Melnikov, E. *Circum-Arctic map of permafrost and ground-ice conditions*. (US Geological Survey Reston, VA, 1997).

[59] Peylin P (2013). Global atmospheric carbon budget: results from an ensemble of atmospheric CO2 inversions. Biogeosciences.

[60] Peters W (2007). An atmospheric perspective on North American carbon dioxide exchange: CarbonTracker. Proc. Natl Acad. Sci. USA.

[61] Peters W (2010). Seven years of recent European net terrestrial carbon dioxide exchange constrained by atmospheric observations. Glob. Change Biol..

[62] Chevallier, F. et al. CO2 surface fluxes at grid point scale estimated from a global 21 year reanalysis of atmospheric measurements. *Journal of Geophysical Research-Atmospheres* **115**, 10.1029/2010jd013887 (2010).

[63] Rödenbeck C, Conway T, Langenfelds R (2006). The effect of systematic measurement errors on atmospheric CO 2 inversions: a quantitative assessment. Atmos. Chem. Phys..

[64] Rödenbeck C, Houweling S, Gloor M, Heimann M (2003). CO 2 flux history 1982–2001 inferred from atmospheric data using a global inversion of atmospheric transport. Atmos. Chem. Phys..

[65] Saeki T, Patra PK (2017). Implications of overestimated anthropogenic CO 2 emissions on East Asian and global land CO 2 flux inversion. Geosci. Lett..

[66] Kolby Smith W (2016). Large divergence of satellite and Earth system model estimates of global terrestrial CO2 fertilization. Nat. Clim. Change.

[67] Pinzon JE, Tucker CJ (2014). A non-stationary 1981–2012 AVHRR NDVI3g time series. Remote Sens..

[68] Heinsch FA (2006). Evaluation of remote sensing based terrestrial productivity from MODIS using regional tower eddy flux network observations. Ieee Trans. Geosci. Remote Sens..

[69] Turner DP (2006). Evaluation of MODIS NPP and GPP products across multiple biomes. Remote Sens. Environ..

[70] Zhao MS, Heinsch FA, Nemani RR, Running SW (2005). Improvements of the MODIS terrestrial gross and net primary production global data set. Remote Sens. Environ..

[71] Ballantyne A (2017). Accelerating net terrestrial carbon uptake during the warming hiatus due to reduced respiration. Nat. Clim. Change.

[72] Pettorelli N (2005). Using the satellite-derived NDVI to assess ecological responses to environmental change. Trends Ecol. Evol..

[73] Reichstein M (2005). On the separation of net ecosystem exchange into assimilation and ecosystem respiration: review and improved algorithm. Glob. Change Biol..

[74] Lasslop G (2010). Separation of net ecosystem exchange into assimilation and respiration using a light response curve approach: critical issues and global evaluation. Glob. Change Biol..

[75] Barr AG (2013). Use of change-point detection for friction–velocity threshold evaluation in eddy-covariance studies. Agric. For. Meteorol..

[76] Sitch S (2015). Recent trends and drivers of regional sources and sinks of carbon dioxide. Biogeosciences.

[77] Jones LA (2017). The SMAP level 4 carbon product for monitoring ecosystem land–atmosphere CO_2_ exchange. IEEE Trans. Geosci. Remote Sens..

[78] Chapin FS (2006). Reconciling carbon-cycle concepts, terminology, and methods. Ecosystems.

[79] Harris I, Jones PD, Osborn TJ, Lister DH (2014). Updated high-resolution grids of monthly climatic observations - the CRU TS3.10 Dataset. Int. J. Climatol..

[80] Obu J (2019). Northern Hemisphere permafrost map based on TTOP modelling for 2000–2016 at 1 km^2^ scale. Earth-Sci. Rev..

[81] Gruber A, Scanlon T, van der Schalie R, Wagner W, Dorigo W (2019). Evolution of the ESA CCI Soil Moisture climate data records and their underlying merging methodology. Earth Syst. Sci. Data.

[82] Fan Y (2016). Applications of structural equation modeling (SEM) in ecological studies: an updated review. Ecol. Process..

[83] Rosseel Y (2012). Lavaan: An R package for structural equation modeling and more. Version 0.5–12 (BETA). J. Stat. Soft..

[84] Piao SL (2017). Weakening temperature control on the interannual variations of spring carbon uptake across northern lands. Nat. Clim. Change.

