# Supplementary material for: Respiratory loss during late-growing season determines the net carbon dioxide sink in northern permafrost regions
Source: Nat Commun. 2022 Sep 26;13:5626. doi: 10.1038/s41467-022-33293-x (PMC9512808; doi:10.1038/s41467-022-33293-x)
Supplement: Supplementary file 1 — Supplementary figures and tables [file 41467_2022_33293_MOESM1_ESM.pdf]

This supplementary file includes:

1. Tables S1
2. Figs. S1 to S15
3. Supplementary References
4. Supplementary text to describe in full detail about using mixed effects model analysis of net CO_2 trends over permafrost and non-permafrost regions using ACIs

Table S1: NHL Eddy Covariance (EC) sites (Fig. S1, n = 48) from FLUXNET2015 used in the analysis. Detailed descriptions of the FLUXNET2015 dataset, including reference for each site, can be found in [1].

SITE_ID	longitude	latitude	IGBP	YRS	MAT	MAP	ELE	%P
CA-Man	-98.4808	55.8796	ENF	15 (1994-2008)	-3.2	520	259	NA
CA-NS1	-98.4839	55.8792	ENF	5 (2001-2005)	-2.89	500	260	NA
CA-NS2	-98.5247	55.9058	ENF	5 (2001-2005)	-2.88	500	260	NA
CA-NS3	-98.3822	55.9117	ENF	5 (2001-2005)	-2.87	502	260	NA
CA-NS4	-98.3806	55.9144	ENF	4 (2002-2005)	-2.87	502	260	NA
CA-NS5	-98.485	55.8631	ENF	5 (2001-2005)	-2.86	500	260	NA
CA-NS6	-98.9644	55.9167	OSH	5 (2001-2005)	-3.08	495	244	NA
CA-NS7	-99.9483	56.6358	OSH	4 (2002-2005)	-3.52	483	297	NA
CA-Oas	-106.198	53.6289	DBF	15 (1996-2010)	0.34	429	530	NA
CA-Obs	-105.118	53.9872	ENF	14 (1997-2010)	0.79	406	628.94	14
CA-SF1	-105.818	54.485	ENF	4 (2003-2006)	0.4	470	536	NA
CA-SF2	-105.878	54.2539	ENF	5 (2001-2005)	0.4	470	520	14
CA-SF3	-106.005	54.0916	OSH	6 (2001-2006)	0.4	470	540	14
DE-Akm	13.6834	53.8662	WET	6 (2009-2014)	8.7	558	-1	NA
DE-Hai	10.453	51.0792	DBF	13 (2000-2012)	8.3	720	430	NA
DE-Lnf	10.3678	51.3282	DBF	11 (2002-2012)	6.96	894.6	451	NA

DE-Obe	13.7213	50.7867	ENF	7 (2008-2014)	5.5	996	734	NA
DE-RuR	6.3041	50.6219	GRA	4 (2011-2014)	7.7	1033	514.7	NA
DE-Spw	14.0337	51.8923	WET	5 (2010-2014)	8.7	558	61	NA
DE-Tha	13.5652	50.9624	ENF	19 (1996-2014)	8.2	843	385	NA
DK-Eng	12.1918	55.6905	GRA	4 (2005-2008)	8	613	10	NA
FI-Lom^{p}	24.2092	67.9972	WET	3 (2007-2009)	-1.4	484	274	NA
FI-Sod	26.6378	67.3619	ENF	14 (2001-2014)	-1	500	180	NA
NL-Hor	5.0713	52.2404	GRA	8 (2004-2011)	10	800	2.2	NA
NL-Loo	5.7436	52.1666	ENF	18 (1996-2013)	9.8	786	25	NA
RU-Che	161.3414	68.613	WET	4 (2002-2005)	-11	197	6	100
RU-Cok	147.4943	70.8291	OSH	12 (2003-2014)	-14.3	232	48	100
RU-Fyo	32.9221	56.4615	ENF	17 (1998-2014)	3.9	711	265	NA
RU-Ha1	90.0022	54.7252	GRA	3 (2002-2004)	-0.07	591.87	446	NA
RU-Sam	126.4958	72.3738	GRA	13 (2002-2014)	NA	NA	NA	100
RU-SkP	129.168	62.255	DNF	3 (2012-2014)	NA	NA	246	100
RU-Tks	128.8878	71.5943	GRA	5 (2010-2014)	-12.7	323	7	100
SE-St1	19.0503	68.3542	WET	3 (2012-2014)	-0.7	303	351	14
US-Atq	-157.409	70.4696	WET	6(2003-2008)	-9.7	93	15	100
US-Ivo	-155.75	68.4865	WET	4(2004-2007)	-8.28	304	568	100

US-Prr	-147.488	65.1237	ENF	4(2010-2013)	-2	275	210	50
BE-Bra	4.51984	51.3076	MF	23(1996-2014)	9.8	750	16	NA
BE-Vie	5.99808	50.3049	MF	23(1996-2014)	7.8	1062	493	NA
DK-Sor	11.64464	55.4858	DBF	23(1996-2014)	8.2	660	40	NA
FI-Hyy	24.29477	61.8474	ENF	23(1996-2014)	3.8	709	181	NA
FI-Let	23.95952	60.6418	ENF	10(2009-2012)	4.6	627	119	NA
FI-Sii	24.19285	61.8326	WET	6 (2004-2010)	3.5	701	NA	NA
FI-Var	29.61	67.7549	ENF	3 (2016-2018)	-0.5	601	NA	NA
SE-Deg	9.556539	64.1820	WET	18 (2001-2018)	1.2	523	NA	NA
SE-Htm	13.41897	56.0976	ENF	4 (2015-2018)	7.4	707	NA	NA
SE-Nor	17.4795	60.0865	ENF	5 (2014-2018)	5.5	527	NA	NA
SE-Ros	19.738	64.1725	ENF	5 (2014-2018)	1.8	614	NA	NA
SE-Svb	19.7745	64.2561	ENF	5 (2014-2018)	1.8	614	NA	NA

Note: IGBP: International Geosphere-Biosphere Programme (IGBP) land cover classifications. YRS: number of years with continuous measurement, including start and end years. MAT: mean annual temperature (°C). MAT: mean annual precipitation (mm yr^{-1}). ELEV: elevation (meters). ENF: Evergreen Needleleaf Forests; NDF: Deciduous Needleleaf Forests; MF: Mixed Forests; OSH: Open Shrublands; WSA: Woody Savannas; SAV: Savannas, GRA: Grasslands; WET: Wetlands; CRO: Cropland. %P: percent of permafrost extent on the EC sites based on ESA CCI permafrost extent data (Fig 1b).

Supplementary figures

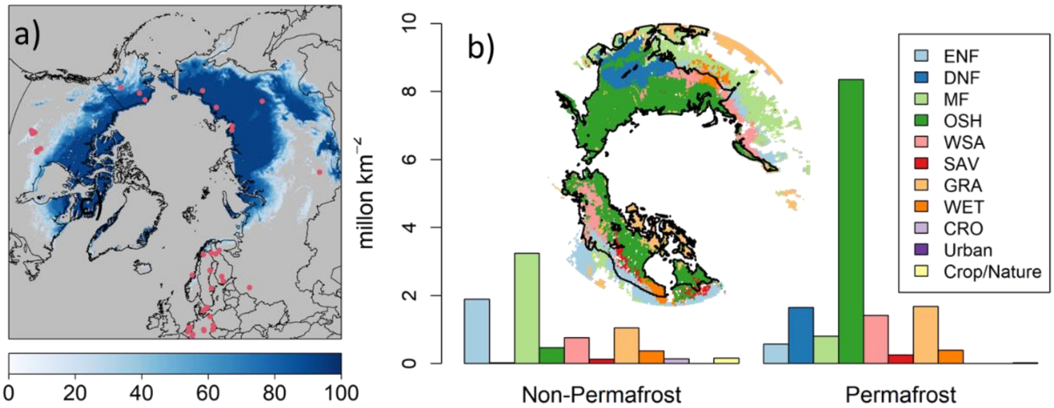

Fig. S1: (a) The spatial distribution of permafrost extent, overlaid with Eddy Covariance (EC) flux tower locations (red dots, n = 48), (b) land cover types based on Moderate Resolution Imaging Spectroradiometer (MODIS) International Geosphere-Biosphere Programme (IGBP) classifications, and land cover statistics in NHL permafrost and non-permafrost regions. ENF: Evergreen Needleleaf Forests; NDF: Deciduous Needleleaf Forests; MF: Mixed Forests; OSH: Open Shrublands; WSA: Woody Savannas; SAV: Savannas, GRA: Grasslands; WET: Wetlands; CRO: Cropland; Urban: Urban and Built-up Lands, Crop/Nature: Cropland/Natural Vegetation Mosaics.

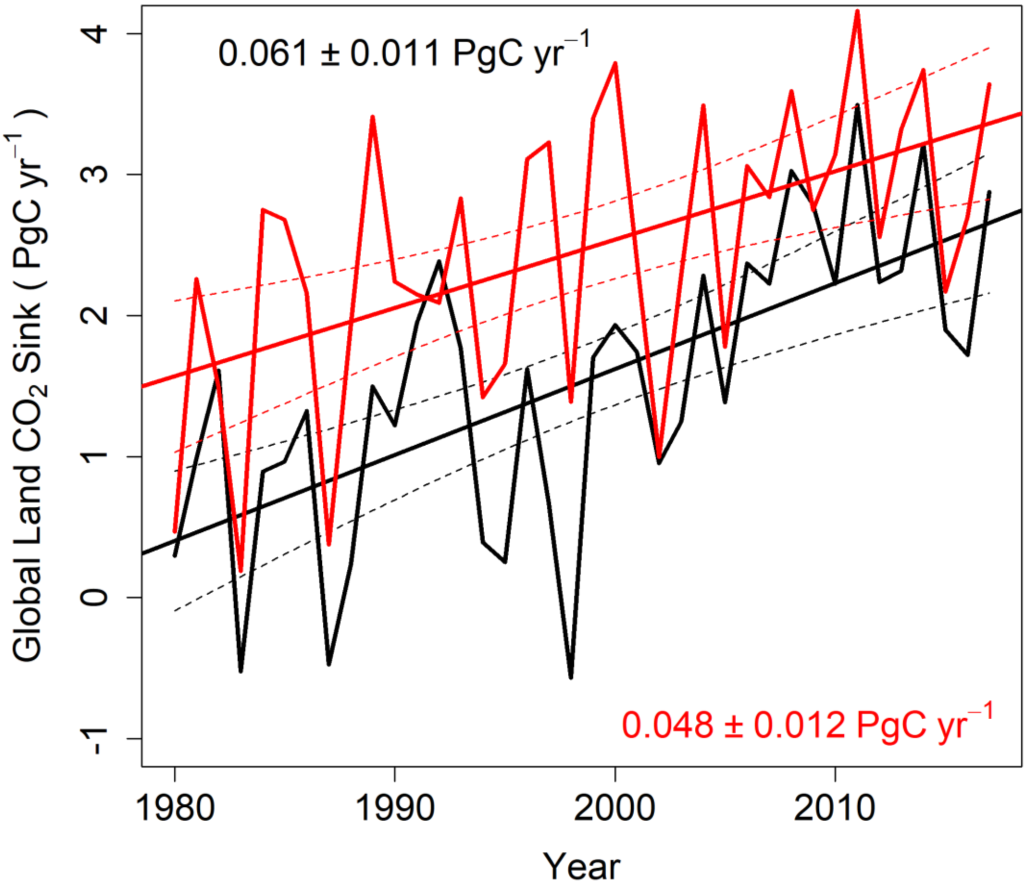

Fig. S2: Comparison of the estimated global land CO_2 sink trend from an ensemble of ACIs (black) and the global CO_2 budget 2020 (GCB2020, red); both assessments are correlated (Pearson's correlation r = 0.79, p < 0.01), while numbers in the figure represent the estimated net carbon uptake trends.

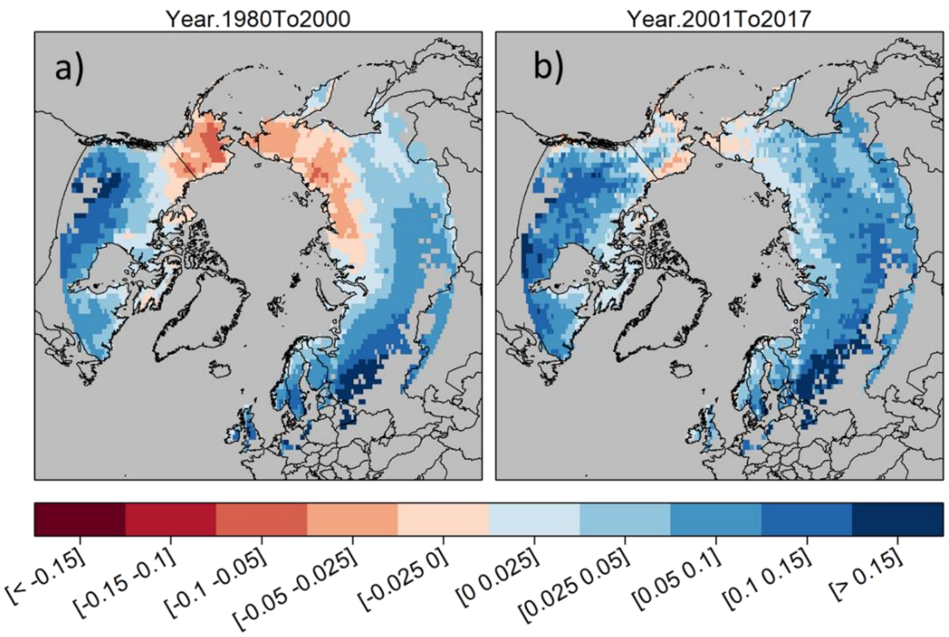

Fig. S3: Spatial patterns of annual net CO_2 uptake (gC m^{-2} d^{-1}) using an ensemble mean of ACIs in the NHL between 1980-2000 (a) and 2001-2017 (b). Here we define the mass balance with respect to the biosphere, such that positive and negative numbers represent ecosystem net CO_2 uptake (blue shades) and losses (red shades). The ACI ensemble includes CarbonTracker (CT2019B), CarbonTracker Europe (CTE2020), CAMS, Jena CarboScope (s76_v4.2 and s85_v4.2), and JAMSTEC.

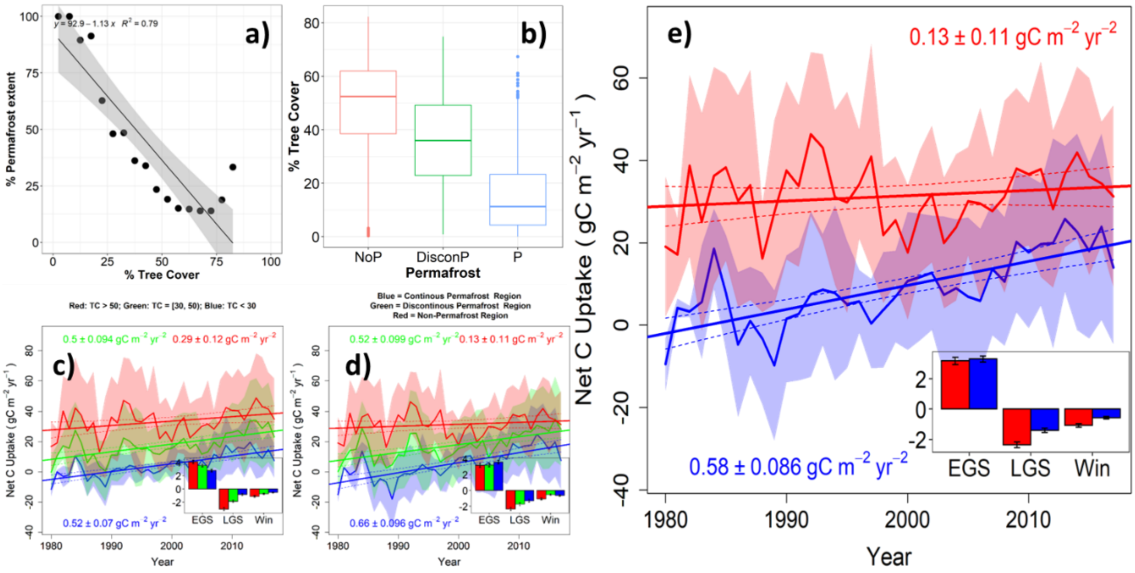

Fig. S4: Correlation between tree cover and permafrost extent at 5% intervals (a) and (b) regional scales. (c) Trends of annual net CO_2 uptake in low (blue color, TC < 30%), intermediate (green color, 30% < TC < 50%), high (red color, TC > 50%) tree cover regions. (d) Trends of annual net CO_2 uptake in continuous permafrost (blue color, P > 90%), discontinuous permafrost (green color, 10% < P < 90%), non-permafrost (red color, P < 10%) regions. (e) Trends of annual net CO_2 uptake in permafrost (blue color, P > 10%) and non-permafrost (red color, P < 10%) regions. in c-e, Numbers represent the net CO_2 uptake trend for each respective. Shading denotes 1 standard deviation (SD) from the 6 individual ACIs. Inset shows clear seasonal trends (gC m^{-2} yr^{-2}) of increasing biospheric net CO_2 uptake in the early-growing season (EGS: May-Aug), and increasing net CO_2 release in the late-growing season (LGS: Sep-Oct) and winter (Win: Nov-Apr).

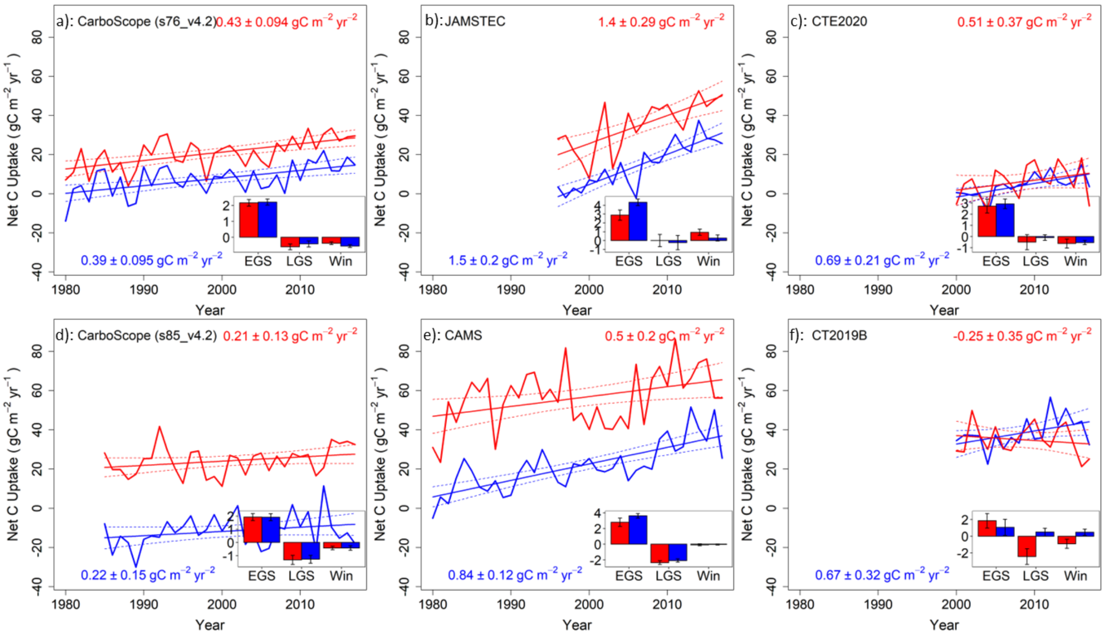

Fig. S5: Trends in net CO_2 uptake for different NHL regions and seasons based on individual ACIs (a: CarboScope s76_v4.2, b: JAMSTEC, c: CTE2020, d: CarboScope s85_v4.2, e: CAMS, f: CT2019B), where permafrost trends are plotted in blue and non-permafrost in red. The dotted lines denote 90% confidence intervals. Numbers in each subfigure represent the net CO_2 uptake trend (mean ± SD) for permafrost (in blue) and non-permafrost (in red) regions. Abbreviations: EGS, early growing season; LGS. late growing season; Win, winter.

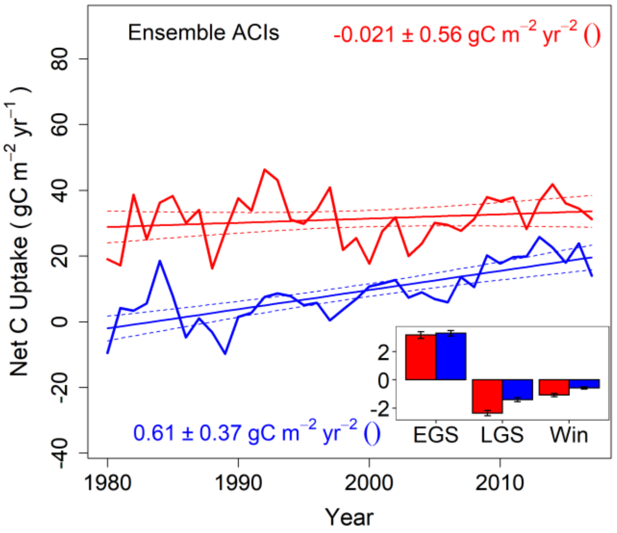

Fig. S6: Trends for net CO_2 uptake for different NHL regions and seasons based on random starting years and length of record (>= 10 years) using the ensemble of ACIs, where permafrost trends are plotted in blue and non-permafrost in red, and dotted lines denote 90% confidence intervals. Numbers represent the net CO_2 uptake trend (mean ± SD) for permafrost (in blue) and non-permafrost (in red) regions. EGS, early growing season; LGS, late growing season; Win, winter.

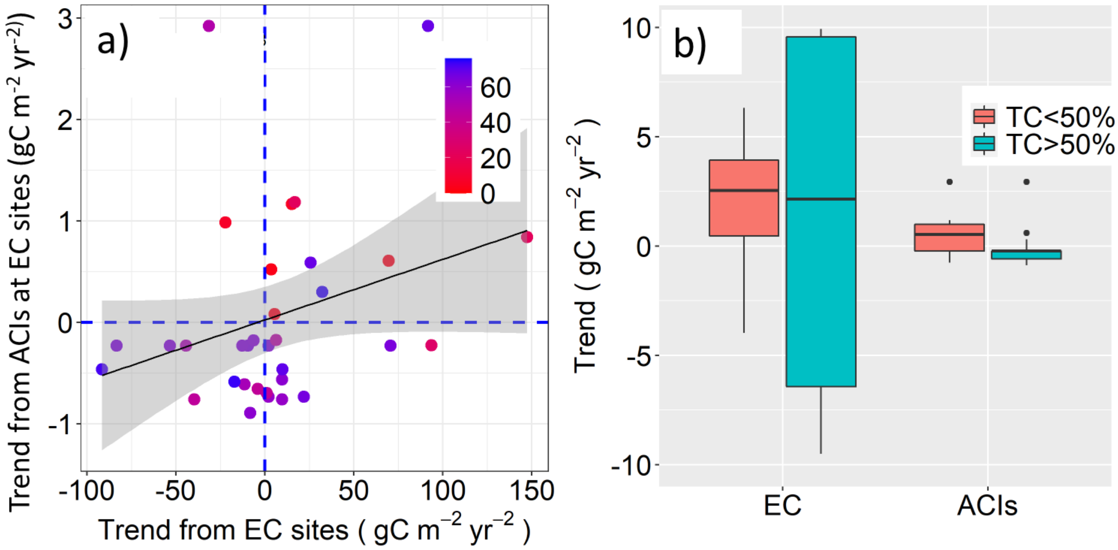

Fig. S7: Site-level comparison of trends using EC and ACIs at the EC site locations. Panel (a) shows the correlation between trends calculated from EC and ACIs at the EC site locations, colored by tree cover. Panel (b) shows the average trends calculated from EC and ACIs at the EC site locations at short-vegetated (TC < 50%) and tree-dominated (TC > 50%) regions.

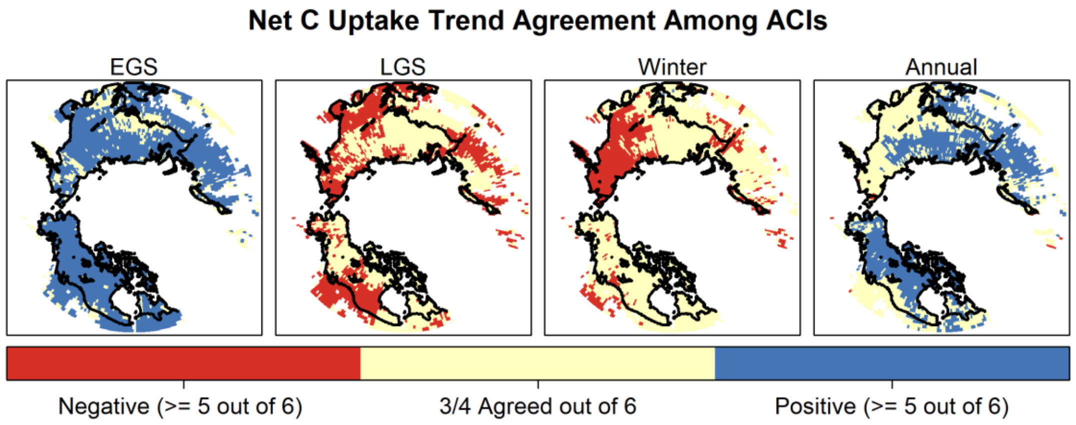

Fig S8. Spatial trend agreement of net CO_2 uptake trends from the different ACIs. In the legend, Negative [**Positive**] indicate at least 5 of 6 ACIs showed decreasing [**increasing**] net CO_2 uptake trends and can be an indicator for high agreement or low uncertain among different inversion data. The 3/4 agreed out of 6 indicates that the only 3 or 4 of 6 ACIs showed a decreasing [**increasing**] net CO_2 uptake trend, and can be an indicator for relatively low agreement or high uncertainty among different inversion data. EGS, early growing season; LGS, late growing season; Win, winter.

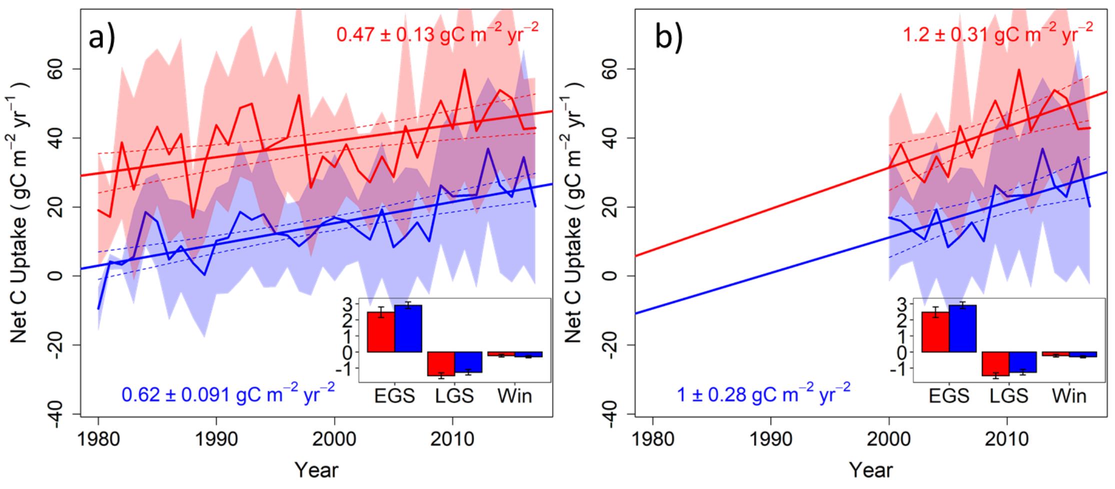

Fig S9. ACI trends of net CO_2 uptake derived using two continuous data records available since 1980 (a, CAMS, and Jena CarboScope (s76)) and ACI data records only available after 2000 (b, CT2020B, CTE2020, and JAMSTEC). Numbers represent the net CO_2 uptake trend (mean ± SD) for permafrost (in blue) and non-permafrost (in red) regions. EGS, early growing season; LGS, late growing season; Win, winter.

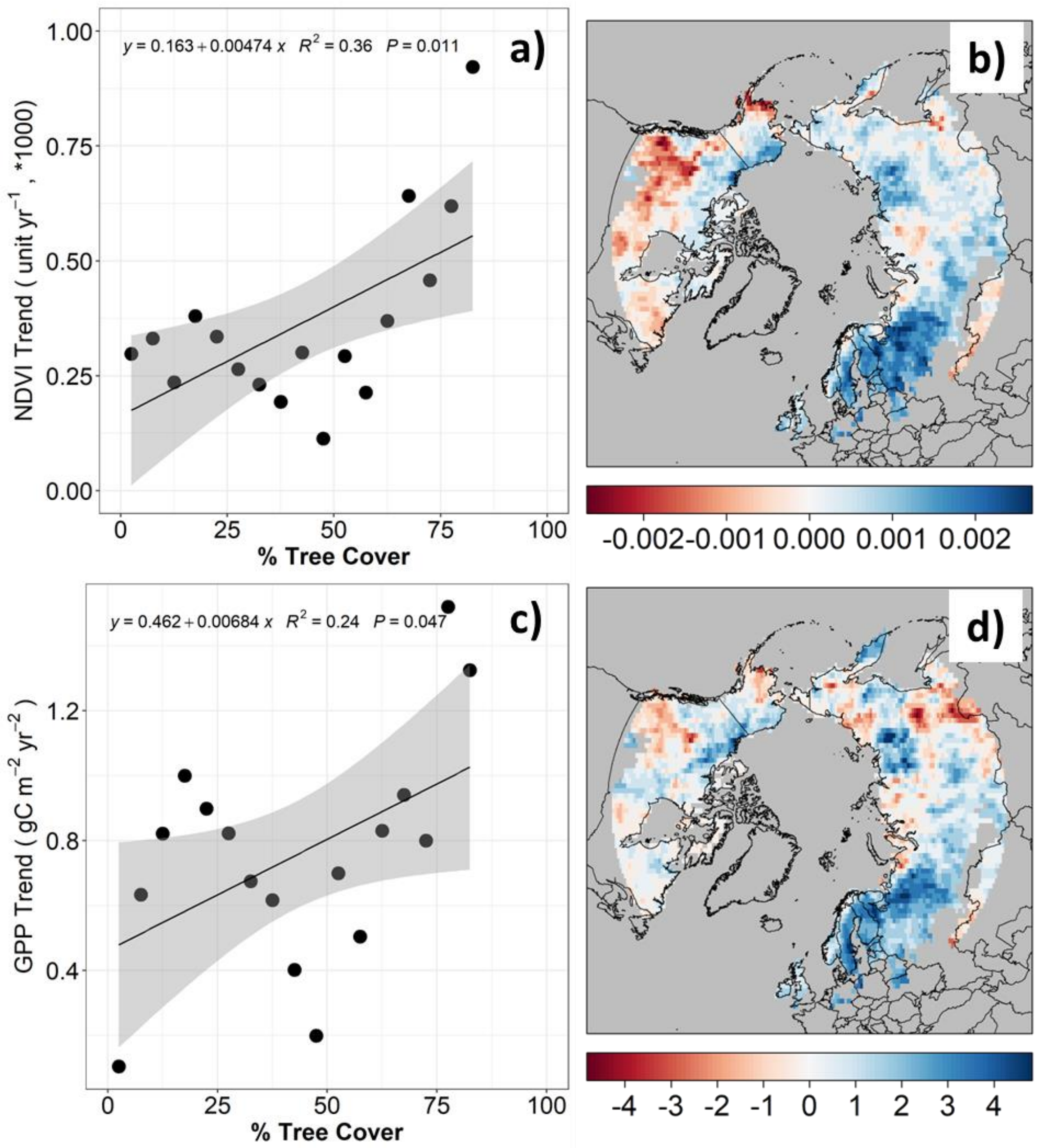

Fig S10. Trend of GPP is increasing faster with higher tree cover, contradicting trends of net CO_2 uptake, and therefore rejecting H1. Panels (a) and (c) are trends of GIMMS NDVI or LUE GPP and along 5% tree cover interval gradient, respectively. Panels (b) and (d) are spatial patterns of trends for GIMMS NDVI or LUE GPP, respectively.

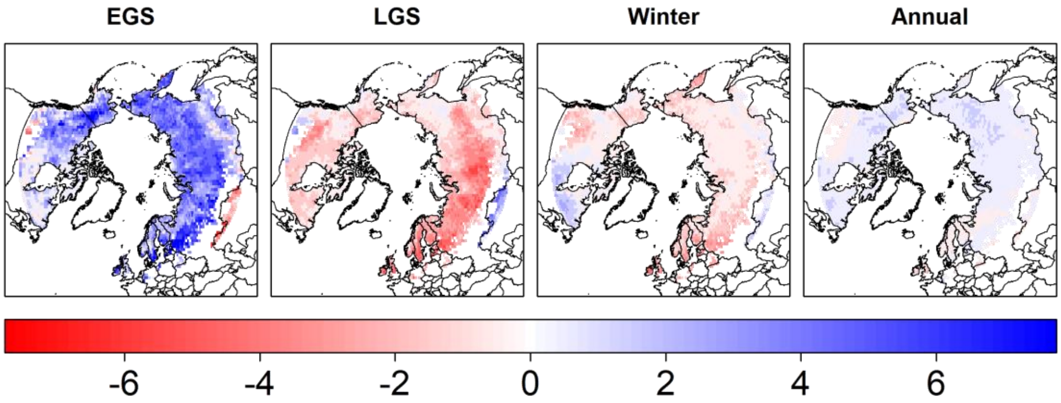

Fig 11. The spatial patterns of trends in net CO_2 uptake (gC $m^{-2}yr^{-2}$) from 1980 to 2017 based on ensemble mean of six ACIs. Positive trends (blue color) indicated enhancement of net CO_2 uptake. Abbreviations: EGS, early growing season; LGS. late growing season.

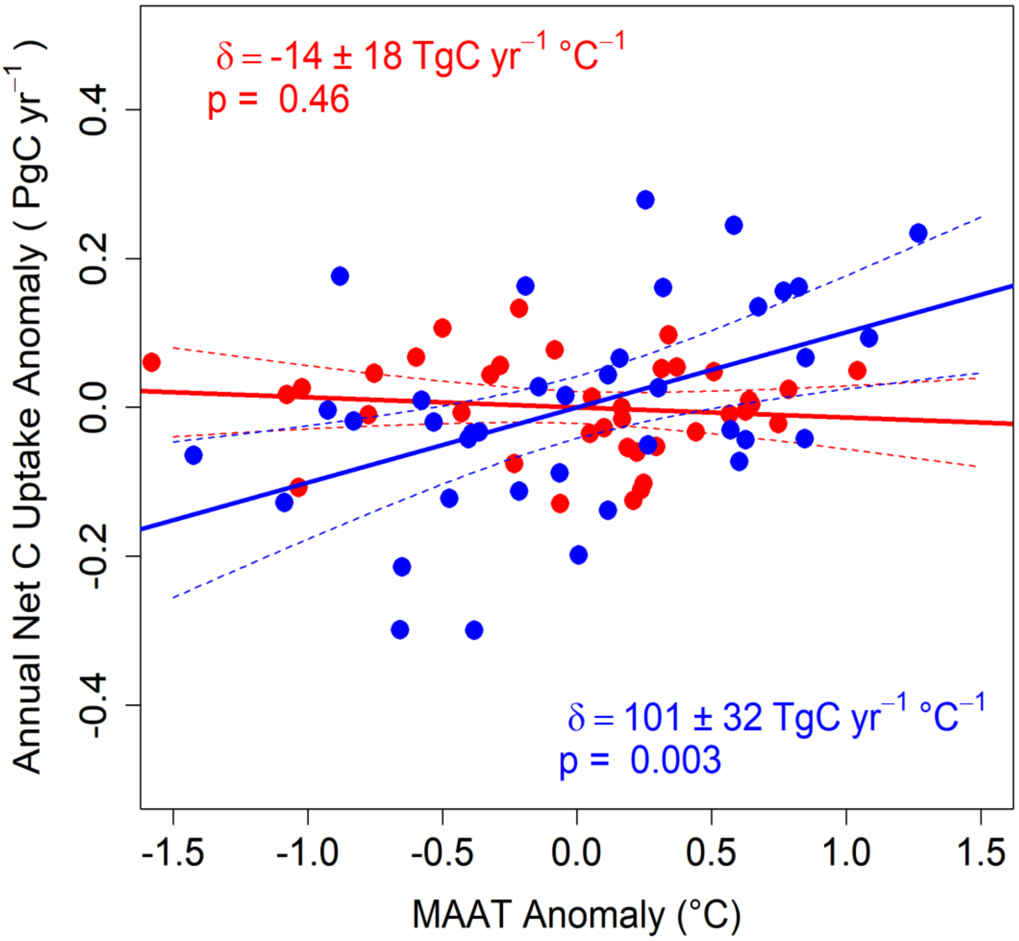

Fig S12 Relationship between annual net CO_2 uptake anomaly and mean annual temperature (MAAT) anomaly for permafrost (blue) and non-permafrost (red) NHL regions. Numbers indicate the temperature sensitivity (TgC yr^{-1} K^{-1}) for the different NHL regions.

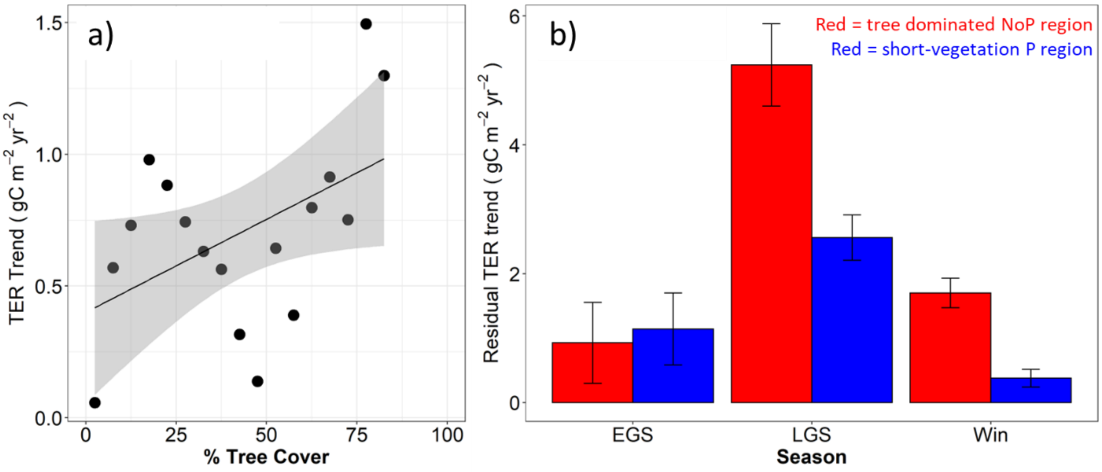

Fig S13 Trends of annual residual toral ecosystem respiration (TER) along the tree cover gradient (a) and seasonal TER for the different NHL regions (b). Abbreviations: EGS, early growing season; LGS. late growing season.

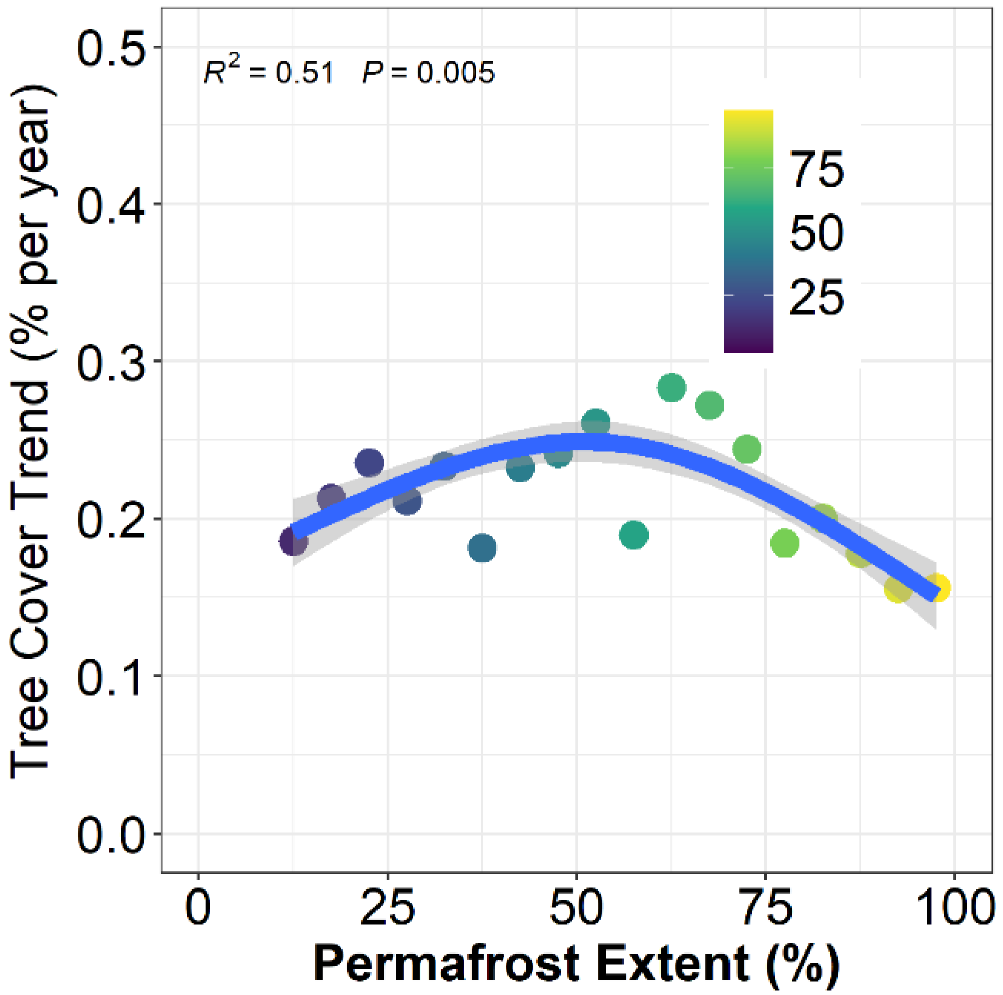

Fig S14 Relationship between tree cover trend (% per year) and permafrost extent (%, right) at 5% intervals, colored by tree cover.

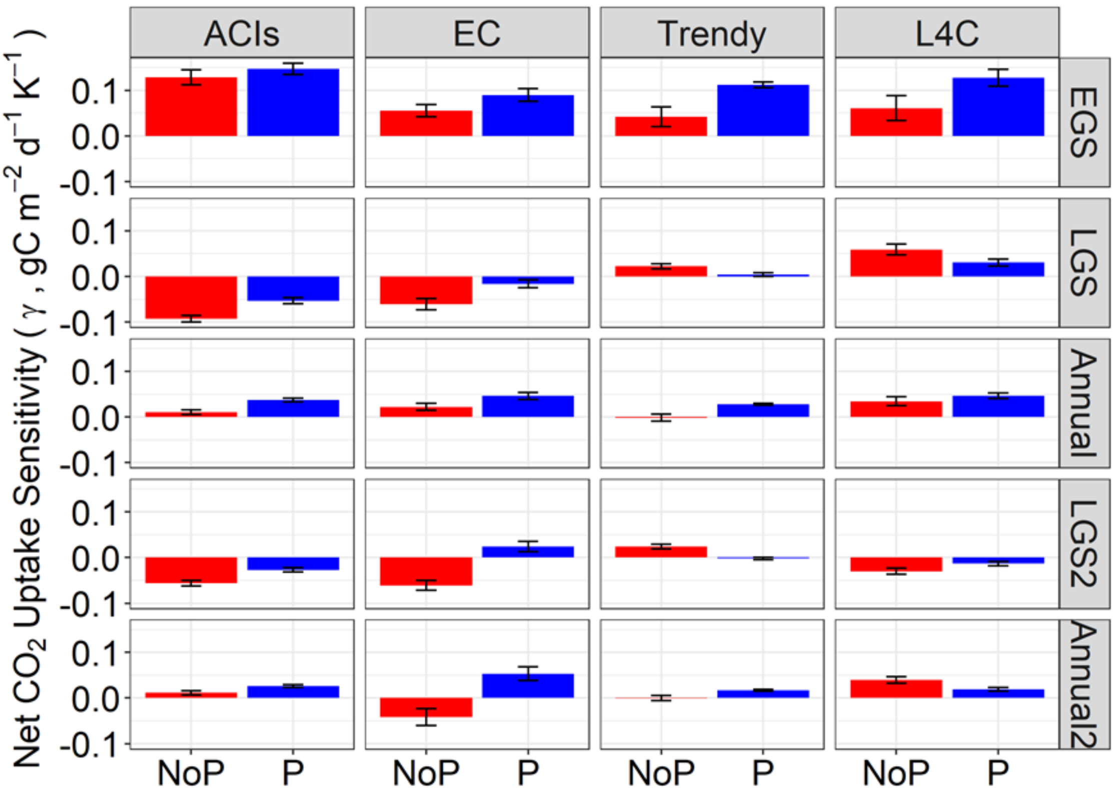

Fig. S15. The direct and lagged effects of temperature to seasonal net CO_2 uptake in short-vegetated permafrost (P) and tree-dominated non-permafrost (NonP) regions using ACIs, EC, and TRENDY assessments. The lagged effects of temperature was quantified by sensitivity of net CO_2 uptake in early growing season (EGS), late growing season (LGS) and annual (Annual) in response to spring (May -June) temperature. The direct effects of temperature were quantified by sensitivity of net CO_2 uptake in late growing season (LGS2) and annually (Annual2) in response to the same season temperature. Sensitivity (γ: gC m^{-2} day^{-1} K^{-1}) is the change in net CO_2 flux (gC m^{-2} day^{-1}) in response to a 1°C temperature change in the Spring.

Supplementary References

1 Pastorello, G. *et al.* The FLUXNET2015 dataset and the ONEFlux processing pipeline for eddy covariance data. *Sci Data* **7**, 225, doi:10.1038/s41597-020-0534-3 (2020).

Supplementary text for

Mixed effects model analysis of net CO_2 trends over permafrost and non-permafrost regions using ACIs

1. ***Understanding the uncertainty in ACI estimates and its effects on trend estimates***

To understand the uncertainty in ACI estimates and its effects on trend estimates, we use the general linear mixed effects model (GLMM) to investigate the uncertainty in ACI estimates from: (1) spread across different ACIs; (2) time-dependent differences in spread across ACI estimates; and (3) differences among ACIs in partitioning of fluxes between permafrost (PF) and non-permafrost (noPF) regions. Instead of using the ensemble mean across ACIs to estimate trends, we use the individual ACI monthly time-series to estimate the trends. Here, we use the regionally-integrated flux (i.e. units in TgC yr^{-1}) to estimate the trend. The areal flux (i.e. units in gC m^{-2} yr^{-1}) produced similar results, assuming no change in areal extent of PF vs. noPF regions (results not shown). First, to understand how these factors (1-3) affect flux estimates, we fit a GLMM model using 6 *ACIs (i)* x 2 PF/noPF *(p)* x (18-38) years *(j)* and consider (1)-(3) as random effects:

$$\text{(1)} \qquad \text{NEE} = \mu + \alpha_i + \beta_{ij} + \gamma_{ip} + \varepsilon_{ijp}$$

Where μ is the average bias (intercept of the model), α_i describes variability across *ACIs* (1 as described above), β_{ij} variability across *ACIs* x *years* (spread may be larger in some years than others, 2 as described above), γ_{ip} is the spread across *ACIs* x *PF* (spread may be related with differences among ACIs in PF/noPF, 3 as described above) and ε_{ijp} is a residual term. These coefficients indicate the relative influence of each factor on the trends estimate.

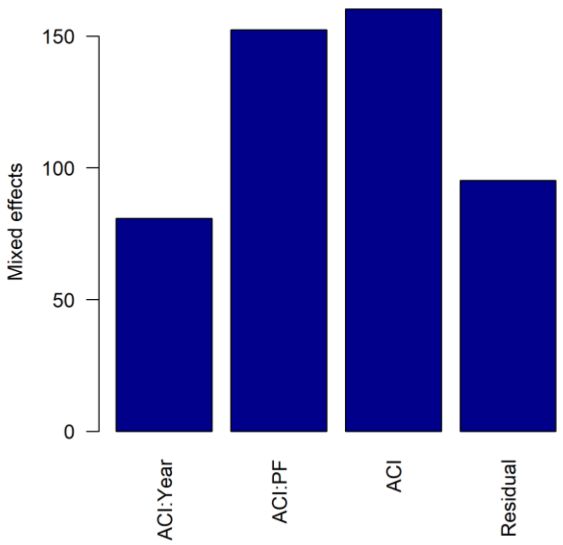

Figure 1. Coefficients for the mixed effects from the GLMM fit in Eq. 1. ACI:Year, ACI:PF, ACI, and residual correspond to β_{ij}, γ_{ip}, α_i, and ε_{ijp} respectively.

Figure 1 suggests variability across *ACI* inversions (1) have the strongest influences on trends, followed by *ACIs* in PF and no PF regions (2) and variability between inversions-years (3), which may indicate inversions differ in the trends. This points to the distinction between PF and no PF regions and temporal variability as important factors explaining variance of net CO_2 trends.

We therefore tested a second model, in which we assumed that differences between *ACIs* can additionally be due to (i) variability between years, including possible trends (δ_j), (ii) differences between PF and no PF regions (ζ_p) and (iii) differences between PF regions and years (κ_{pj}):

$$\text{(2)} \quad NEE = \mu + \alpha_i + \beta_{ij} + \gamma_{ip} + \delta_j + \zeta_p + \kappa_{pj} + \varepsilon_{ijp}$$

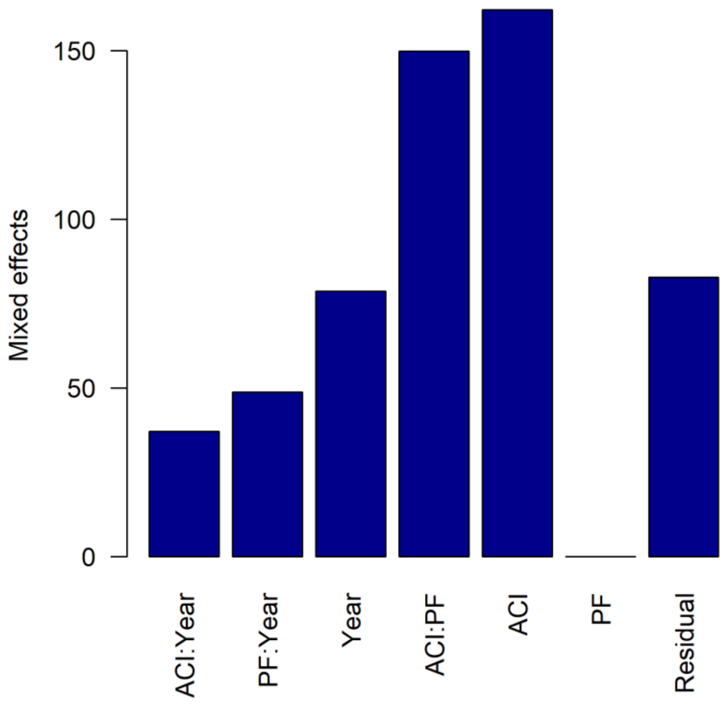

Figure 2. Coefficients for the mixed effects from the GLMM fit in Eq. 2. ACI:Year, PF:Year, Year, ACI:PF, ACI, PF, and residual correspond to β_{ij}, κ_{pj}, δ_j, γ_{ip}, α_i, ζ_p, and ε_{ijp} respectively.

The model from *Eq. 2* provides a significantly better fit to the data than the model from *Eq. 1.*, given by ANOVA analysis (R package anova). The *Eq. 2* model conditional R^2 is 0.83.

Coefficients from *Eq. 2* suggest that, in addition to 1-3, temporal variability in NEE (δ_j) and differences between PF regions and years (κ_{pj}) are also important contributors to trend estimates.

Finally, we define a set of models with different combinations of the two fixed and random effects explored before. We start with the full model (*Eq. 3.*) and select the model that provides the best fit based on the conditional R^2, using ANOVA.

$$\text{(3)} \qquad NEE = aX_j + bX_{pj} + \mu + \alpha_i + \beta_{ij} + \gamma_{ip} + \delta_j + \zeta_p + \kappa_{pj} + \varepsilon_{ijp}$$

$$\text{(4, best model)} \qquad NEE = aX_j + \mu + \alpha_i + \beta_{ij} + \gamma_{ip} + \delta_j + \zeta_p + \kappa_{pj} + \varepsilon_{ijp}$$

Where X_j is time in years and *a* corresponds to the NEE trend, and *b* expresses an interaction term between trend and PF/noPF. The ANOVA indicates that *Eq. 4.* is significantly better (conditional R^2 =

0.92) than the full model (*Eq. 3.*, conditional $R^2 = 0.74$). These analyses indicate that IAV or spread across different ACIs, and differences between PF and no PF regions have strong influences on net CO_2 trends.

2. Comparing trend estimates from different models

The previous analysis supports an important role of PF presence and differences in trends in permafrost and non-permafrost (PF vs. NoPF) regions. Therefore, we compare the best performing GLMM models (*Eq. 4*) with those from simple linear regression model (LRM) fits to the dataset with individual ACIs (*Eq. 5*), as well as with the ensemble mean of ACIs (*Eq. 5*) to see if different models produce significantly different trend estimates:

(5) NEE = aX_{ij} (LRM, using individual monthly ACI time-series)

(6) NEE = aX_j (LRM, using ensemble mean monthly ACI time-series)

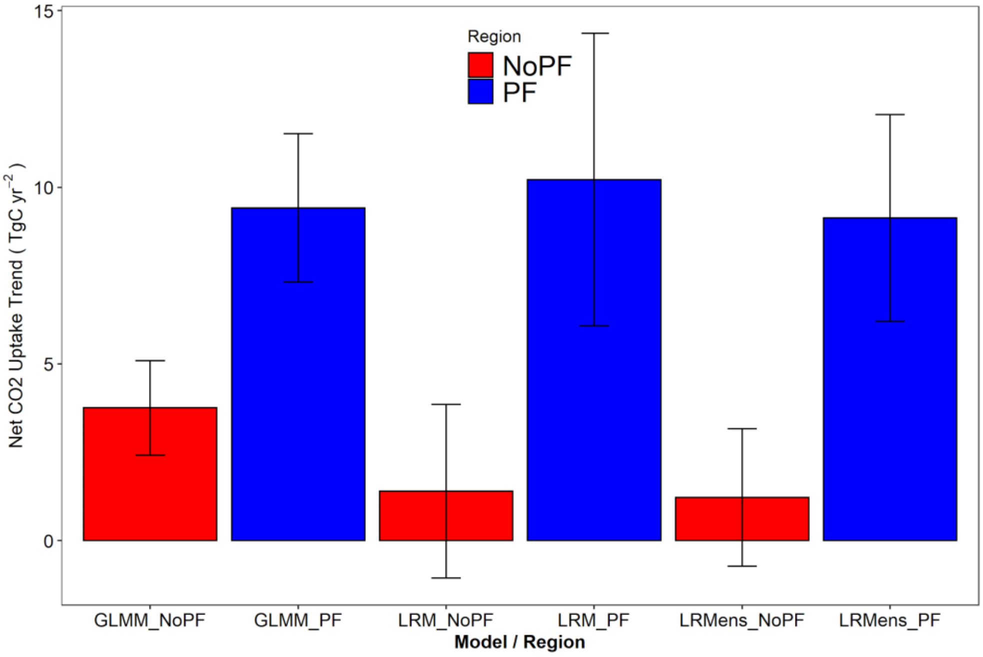

Figure 3. Trends estimated separately for permafrost (PF; blue) and non-permafrost (NoPF; red) regions, including random effects from ACI uncertainty (GLMM, E.q. 4), compared to results from the LRM using individual ACIs (LRM, Eq. 5) or the ensemble mean of ACIs (LRMens, Eq. 6) .

Figure 3 shows that after accounting for the uncertainty in ACIs, the trend of net CO_2 uptake in permafrost regions (i.e. GLMM_P) is comparable to simple linear regression (i.e. LRM_P and LRMens_P). The trend of net CO_2 uptake in non-

permafrost regions is slightly higher after accounting for uncertainty in ACIs (i.e. GLMM_NoP), but is not significantly different from the linear regression models (LRM_NoP and LRMens_NoP). However, all models show that the trend of net CO_2 uptake is significantly higher in permafrost regions than non-permafrost regions, and there is no significant difference in net CO_2 uptake trends across different models.

Therefore, after considering multiple sources of uncertainty affecting the ACI estimates in our GLMM approach, the difference between trends in permafrost and non-permafrost regions is slightly reduced, but the trends remain significantly different.
