# Supplementary material for: Respiratory loss during late-growing season determines the net carbon dioxide sink in northern permafrost regions
Source: Nat Commun. 2022 Sep 26;13:5626. doi: 10.1038/s41467-022-33293-x (PMC9512808; doi:10.1038/s41467-022-33293-x)
Supplement: Supplementary file 2 — Peer Review File [file 41467_2022_33293_MOESM2_ESM.pdf]

nature portfolio

Peer Review File

Respiratory loss during late-growing season determines the net carbon dioxide sink in northern permafrost regions

Open Access This file is licensed under a Creative Commons Attribution 4.0 International License, which permits use, sharing, adaptation, distribution and reproduction in any medium or format, as long as you give appropriate credit to the original author(s) and the source, provide a link to the Creative Commons license, and indicate if changes were made. In the cases where the authors are anonymous, such as is the case for the reports of anonymous peer reviewers, author attribution should be to 'Anonymous Referee' followed by a clear attribution to the source work. The images or other third party material in this file are included in the article's Creative Commons license, unless indicated otherwise in a credit line to the material. If material is not included in the article's Creative Commons license and your intended use is not permitted by statutory regulation or exceeds the permitted use, you will need to obtain permission directly from the copyright holder. To view a copy of this license, visit http://creativecommons.org/licenses/by/4.0/.

Reviewer comments, first round review:

Reviewer #1 (Remarks to the Author):

The authors use two empirical data streams (atmospheric CO_2 concentrations and eddy covariance) to estimate trends (1980-2017) in terrestrial carbon uptake across latitudinal gradients in permafrost and non-permafrost regions. They then contrast the results from these empirical estimates to model outputs for an ensemble assessment (TRENDY) and use structural equation modeling to estimate the drivers of trends for the three assessments of C uptake for different seasons. The objectives of this research were to (1) quantify latitudinal trends and drivers of CO_2 uptake for permafrost and non-permafrost regions for different seasons and (2) determine the climatic and vegetative drivers of these trends. The drivers the authors explored are common measurements made through remote sensing and regional climatic estimates. An important focus of this research is the differentiation between early growing season, late growing season and winter fluxes as fluxes during these times of year can have a disproportionate effect on annual C balance. Overall, it is an impressive effort.
Strengths
The paper merges a robust number of datasets in an ensemble analysis that is interesting and timely.
Documenting latitudinal response to climatic variables using different data streams, and for different seasons, is novel.
Comparison to model output for different times of season.
Weakness
Use of permafrost-presence as a defining characteristic creates a confusing narrative.
Lack of linkage of results to vegetation characteristics or land use.

Major concerns
1) Permafrost designation: My biggest concern with this paper is the permafrost and non-permafrost area designation, which I think creates issues related to framing the results (below) and an assumption of permafrost as a driving variable. The entire discontinuous/sporadic region is treated as 'permafrost' when it is actually a matrix of permafrost and non-permafrost underlain ecosystems. There are maps that delineate the discontinuous, sporadic and continuous zones--- Why not use these? I suspect that three permafrost categories for the ACI (perm, no perm, and a grouped discontinuous/sporadic perm) would provide stronger support for the differences in late-, early- and annual flux trends.

A similar concern for the eddy covariance. For instance, I happen to know that several of the eddy-covariance sites that have 'p's' next to them (Table S1) to designate permafrost do not (or did not at the time of measurement) have permafrost in the upper 1 meter. This is a problem at a pretty fundamental level—accurately describing the data in one's analysis. I suggest the authors contact the individuals responsible for all of their sites and ask if they have permafrost within the soil profile (to a depth that is biologically meaningful) or published soil temperatures.

Regardless of whether the authors better estimate permafrost zonation, I believe trying to frame the results as a 'permafrost vs. non-permafrost' regional phenomenon creates a confusing narrative that may the miss the actual mechanisms driving the documented variation in net CO_2 uptake occurs. Specifically, I think the relative potential vegetation response to climate varies across the region and this is likely is the driver of the trend differences. Tundra shrubs often have a greater capacity to respond to climate than do tree species and this might be the reason for these results (see below).

2) Vegetation cover: Typically in non-permafrost regions of the far northern high latitudes the dominant vegetation is continuous forest cover. Trees can extend from low latitudes (52-60 ºN to very high latitudes (60-70 ºN) in non-permafrost regions. However, in the so called 'permafrost zone' of this paper there is a more consistent transition that occurs from trees, to shrubs, to tundra (e.g. graminoid) across most of the studied areas. I expect that the strength of the C uptake response reflects this gradient more than anything related to permafrost presence

(including NE Siberia where deciduous larch allows for a vigorous shrub presence). Shrub expansion both in terms of profile (height, density) and coverage influences snow dynamics in the shrub-to-tundra transitional areas. This feeds back onto the seasonality of flux dynamics as it influences snow accumulation and loss. This is really well-documented in the northern high latitudes (some of the authors have published on the topic).

3) Forest land use/disturbance: Using 52ºN latitude cutoff, then a significant fraction of the land cover in the non-permafrost is heavily affected by forest management (harvesting), and is more influenced by forest fires and insect epidemics (e.g. mountain pine beetle) relative to the non-permafrost areas. Some of it is mixed-agriculture and forest, again, unlike permafrost regions. I think the authors should address these issues in the manuscript or supplementary material.

Potential solution
I think this article would be more informative if permafrost was treated as an independent variable in the SEM rather than a definitive category. The authors could assign potential percentage area of permafrost for different coverage (e.g., sporadic) grids based on permafrost distribution maps. The current framing effectively assumes that permafrost presence is continuous in all zones and causative, which is either inaccurate or disputable. If the authors could include vegetation cover type, maybe land use/disturbance (less important) and permafrost presence in their SEM modeling, then I think this would be an incredible paper. As it is currently framed, I think it is creating more confusion than clarity.

Minor concerns
There are a large number of spacing issues, very often in how units are displayed, and other small editorial issues. I did not go through and correct these.

Fig. 4. With a little movement of the numbers in the graph denoting the relationships, all of the correlation values could be fully visible.

Reviewer #2 (Remarks to the Author):

This study analyzed multiple constraints on estimates of carbon cycling across the northern high-latitude region (>50 deg N) from atmospheric inversions, a terrestrial biosphere model ensemble, and flux tower observations and found an agreement in a trend of increasing annual net CO_2 sink strength on land since the 1980s due to increasing uptake by plants outpacing increases in late season respiration.

General comments

This paper outlines a pretty extensive analysis of multiple data sources and models to diagnose and attribute trends in high latitude land carbon cycling. There is a mostly clear overall story, even if it deviates in various ways from the hypotheses and results of other studies. There are a lot of pieces packed into this manuscript, and generally the presentation of such could use some streamlining and clarity particularly with how this study’s results may change (or not) the narrative developing from these other studies. Specific to this paper, though, there are many interesting results that come from some rather clever analyses that together will make a valuable contribution to the literature around these critical issues.

But again, there is so much happening here that it can be hard to follow the main storyline, especially when the onus is on the reader to go to the supplemental nearly every step of the way to find the relevant details of where this information comes from and how these conclusions were arrived at. I would recommend that, in a revised version, the authors consider places where the story could be streamlined while at the same time adding a few key details in particular places so that there is enough explanation and justification in the main text so that the reader does not constantly have to refer to the supplemental material or try to interpret the incredible amount of information packed into the (25!) supplemental figures.

Having said that, the flow of the investigation is logical, the analyses robust, and the methods defensible. It is just a lot of each!

Some general considerations with respect to interpreting the results:
-There's a lot of spatially variability and finer-scale heterogeneity in the continuous-discontinuous-sporadic-isolated permafrost gradient. It seems that those were lumped pretty broadly here, or was the finer-scale heterogeneity maintained?
- How spatially-resolved are the atmospheric inversions with respect to their ability to separate fluxes among these regions?
- How meaningful is the "non-permafrost" region that is arbitrarily >50 deg N but not in the permafrost zones? What about differences in PFT distributions occurring within and between permafrost vs. non-permafrost regions?
- How consistent are the measurements and inversions over this long time period (1980-2017) in terms of methodology, especially the density and spatial distribution of observations (and comparing between early vs. later C balance estimates such as in lines 101-102)? Although I suppose all of this explanation is packed into supplementary text, as alluded to in lines 105 - 119 (which is a lot to digest, but…).
- And while this is touched on in sort of a hypothesis discussion near the end, the authors should consider more fully addressing the role (or lack thereof) that disturbances (particularly fire emissions and permafrost thaw) play in their study's results and especially interpreting them relative to the findings of other studies.

Specific comments
38, 42, 92, 99, 122-124, Figure 1, etc. Change yr-2 to yr-1?
48 & 81. How is compensation(s) being used? Offsets? Balance? Fluxes?
67. Consider using AIMs, which seems more standard?
76. emerge
79. were
87. Note that it is 'Materials and Methods' section in the Supplemental Material document
91-97. Please state the time period for this analysis, along with the areas associated with each the permafrost and non-permafrost regions
93. As with the mean and trend, also include the value for the interannual variability?
154-156 "increasing CO2 uptake in the EGS are similar between the non-permafrost and permafrost regions" (see also 179-180)
189. This idea of "legacy effects" is interesting and worth thinking about and possibly exploring further. However, it is sort of just mentioned as a one-off thought here and not really explained - nor defined, i.e. how long of time periods did you look for these effects? Just within the season? Another potential longer-term effect that has been hypothesized is the stimulation of photosynthesis (in the following EGS, and in future years) when more N becomes available as a result of increased Rh in the LGS.
201-205 This translation of your results to recommendations for modelers is great!
206 the acronym SEMs may not be needed here since it is not used again; however, it might be useful to provide a brief explanation of SEMs here, at least in the context of why and how you used the approach in this analysis.
223-234 "in the LGS"
227 re: 'net CO2 uptake in the LGS', I may be misinterpreting this but I was under the impression that there was net release in the LGS - just less than the magnitude of uptake in the EGS? (e.g. see lines 39-40, 157-158, 230)
245-247 I understand that that there's a lot going on in this study and much of the detail is in the supplemental, but this is an example where it feel like at least a mention of the particular satellite system / data set used would be useful to include in the main text; e.g. 'SM' (soil moisture? Acronym not defined earlier. What data set was used? What NDVI data set was used? Time scale? Spatial resolution?)
247-248 'net uptake through respiration'?
267 change 'indicates o' to "identifies"
274 this statement may need just some context or justification, but the wording implies that permafrost regions are sources(?) that have 'switched' to sinks but it hasn't shown up in the observational record. That is not something that your results show (unless I am missing it?) i.e. that they've always been sinks and have increased as such over your time period of analysis. Does

that idea come from another study / studies? If so, when did this switch happen, relative to the time period of your analysis?

281-285 this part now seems to go the opposite way as you postulate that they could switch (again?) to sources due to fire, thaw, etc. But your results showing increased net uptake does not seem to suggest this, not to mention we assume (but not sure?) that the models may or may not handle fire, disturbance, thaw. This has been raised in many other studies, though, so perhaps citing some of those here and explaining how they do or do not jive with your results would be helpful here to clarify what you are trying to convey to the reader with these statements.

312-331 There are a lot of big ideas and concepts stuffed into each paragraph, even each sentence. Consider prioritizing some of these hypothesis, synthesizing a broader perspective, and/or adding some explanation or context where needed (e.g. ‘SOC thermal coupling’ - what does that mean and how does it relate to your study?)

319 should you still use the ‘LGS’ acronym here?

326 why are you focusing now on just the boreal forest?

332 does ‘as a whole’ mean geographically? and/or being comprehensive of all the budget and flux components (e.g. are you including fire in this statement?)

387. “...according to the ensemble of ACIs…”

REVIEWER COMMENTS

Reviewer #1 (Remarks to the Author):

The authors use two empirical data streams (atmospheric CO2 concentrations and eddy covariance) to estimate trends (1980-2017) in terrestrial carbon uptake across latitudinal gradients in permafrost and non-permafrost regions. They then contrast the results from these empirical estimates to model outputs for an ensemble assessment (TRENDY) and use structural equation modeling to estimate the drivers of trends for the three assessments of C uptake for different seasons. The objectives of this research were to (1) quantify latitudinal trends and drivers of CO2 uptake for permafrost and non-permafrost regions for different seasons and (2) determine the climatic and vegetative drivers of these trends. The drivers the authors explored are common measurements made through remote sensing and regional climatic estimates. An important focus of this research is the differentiation between early growing season, late growing season and winter fluxes as fluxes during these times of year can have a disproportionate effect on annual C balance. Overall, it is an impressive effort.

Strengths

The paper merges a robust number of datasets in an ensemble analysis that is interesting and timely.

Documenting latitudinal response to climatic variables using different data streams, and for different seasons, is novel.

Comparison to model output for different times of season.

Response: Thank you for your comments.

Weakness

Use of permafrost-presence as a defining characteristic creates a confusing narrative.

Lack of linkage of results to vegetation characteristics or land use.

Response: Thank you for your constructive comments, that helped us to improve the quality and clarity of this work. In this revision, we kept the strengths of the work, but addressed the various weaknesses following reviewers' suggestions; Below is a summary of the major changes:

(1) We used a finer zonation scheme, based on percent tree cover (%TC) and percent permafrost extent (%P), to study the seasonal dynamics of net CO_2 uptake along both the vegetation and permafrost gradients, and explain the underlying mechanisms. Importantly, we found that vegetation has a stronger control on net CO_2 uptake than permafrost presence and temperature, suggesting a mechanistic link between vegetation and carbon cycle in the northern high latitude region. Therefore, we focused on analyzing how seasonal carbon cycle characteristics related to vegetation (i.e., tree cover) and clarified the underlying mechanisms in the NHL.

(2) We restructured the manuscript in a hypotheses-driven manner, and focus on explaining climatic, vegetation, and environmental controls on the seasonal carbon cycle in the analysis. We also tried to keep the manuscript as concise, clear, and logically structured as possible, which excluded some secondary analyses that were less pertinent to the main points of the study. Please also see our response to Reviewer 2 for more details.

We believe this study provides novel and nuanced insights into our understanding of carbon-vegetation-permafrost interactions in the northern high latitudes, particularly in the context of understanding increasing carbon uptake and CO_2 seasonal amplitude in a warming climate. Please see point by point response below for more details.

Major concerns

1) Permafrost designation: My biggest concern with this paper is the permafrost and non-permafrost area designation, which I think creates issues related to framing the results (below) and an assumption of permafrost as a driving variable. The entire discontinuous/sporadic region is treated as 'permafrost' when it is actually a matrix of permafrost and non-permafrost underlain ecosystems. There are maps that delineate the discontinuous, sporadic and continuous zones---Why not use these? I suspect that three permafrost categories for the ACI (perm, no perm, and a grouped discontinuous/sporadic perm) would provide stronger support for the differences in late-, early- and annual flux trends.

Response: This is a valid concern. To address this concern, we studied the trends in net CO_2 uptake and how they relate to climate, vegetation, and environmental factors along continuous landscape vegetation and permafrost gradients, as well as at broader regional scales.

(1) Analysis along vegetation, climate, and permafrost gradients (lines 194-205 in Material and Methods)

We binned continuous estimates of %TC and %P into 5% intervals, and annual mean air temperature into 1-degree intervals. The net CO_2 uptake for early-growing season (EGS: May-August), late-growing season (LGS: September – October), winter (November-April), and annual periods from 1980 to 2017 was summarized using the ensemble mean of the ACIs (NEEACI) at each binned interval. Then, the seasonal and annual mean net CO_2 uptake for each interval of %TC, %P, and temperature was regressed against years using linear regression. The slope of the regression was interpreted as the net CO_2 uptake trend (gC m^2 yr^{-2}). Trends of net CO_2 uptake at seasonal and annual scales were plotted against %TC, %P, and air temperature to understand the trend and seasonality of net CO_2 uptake along the vegetation (tree cover), climate, and permafrost gradients (Fig 1 and 3).

We found %TC has the strongest controls on seasonal net CO_2 uptake, suggesting a mechanistic link between vegetation and carbon cycle. Therefore, we reported the relationship between tree cover and net CO_2 uptake (Fig 1-4) and added tree cover and permafrost as additional variables

to better explain the mechanisms underlying the seasonal dynamics of net CO_2 uptake in the structural equation modeling (SEM) analysis in this revision.

(2) Regional analysis (lines 206-220 in Material and Methods)

To reduce the pixel-scale uncertainties of net CO_2 uptake using ACIs data, we also calculated the trends of net CO_2 uptake at regional scales, which were classified by %TC and %P (Fig S4). Using %TC, the NHL was divided into low (< 30%), intermediate (30 – 50%), and high (> 50%) tree cover regions. Using %P, the NHL was divided into continuous (ConP, %P > 90%), discontinuous (DisconP, 10% < %P < 90%), and non-permafrost (NoP, %P < 10%) regions following reviewer recommendations. The %TC and %P variables are highly correlated, such that short-vegetation regions (TC < 50%) are primarily in the permafrost region (ConP and DisconP), while tree dominated regions (TC > 50%) are primarily in the non-permafrost region (Fig S4). Spatial and temporal patterns for the net CO_2 uptake trend were calculated seasonally and annually from 1980 to 2017 using the ensemble mean of the ACIs (NEE_{ACI}) over the different NHL regions. Seasonal and annual mean net CO_2 uptake for each region was regressed against years using linear regression. The slope of the regression was interpreted as the net CO_2 uptake trend (gC m2 yr-2, Fig S4).

Since trends of net CO_2 uptake are not statistically different between low tree cover (< 30% tree cover in ConP regions) and intermediate tree cover (30-50% tree cover in DisconP regions), we aggregated these two regions into a short-vegetated (TC < 50%) permafrost region. We contrasted the net CO_2 uptake between short-vegetated (TC < 50%) permafrost and tree-dominated (TC > 50%) non-permafrost regions (lines 222-229 in Material and Methods, lines 135-143 in main text).

While our revised analysis largely confirms the net CO_2 uptake trend across the permafrost-vegetation gradient, it also highlights more nuanced responses and thresholds that can be tested by hypotheses.

A similar concern for the eddy covariance. For instance, I happen to know that several of the eddy-covariance sites that have 'p's' next to them (Table S1) to designate permafrost do not (or did not at the time of measurement) have permafrost in the upper 1 meter. This is a problem at a pretty fundamental level—accurately describing the data in one's analysis. I suggest the authors contact the individuals responsible for all of their sites and ask if they have permafrost within the soil profile (to a depth that is biologically meaningful) or published soil temperatures.
Response: Sorry for the confusion.

In the previous version, we determined the permafrost status of an EC site based on a categorical permafrost zone map from the IPA (International Permafrost Association). Because we lumped sporadic (10-50%), discontinuous (50-90%) and continuous permafrost (90-100%) zones into one permafrost zone (>10%), some EC sites in the sporadic and discontinuous

permafrost regions were labeled as permafrost (P) region. However, some EC sites may be located in well-drained landscapes over sporadic and discontinuous permafrost regions, with active layer thickness (ALT) well over 1 meter. Therefore, these EC sites should not be considered as permafrost sites by some experts due to their deep ALT. We believe this is the source of confusion and apologize for this.

In this revision, we used percent of permafrost extent (%P) to determine permafrost status for each EC site based on a finer delineation permafrost extent dataset (see lines 159-175 in Materials and Methods and Table S1). Therefore, each EC site was assigned to a %P class, rather than discrete permafrost category (Table S1). The EC data was used mainly to (1) do a site-level comparison of the trend of net CO_2 uptake between ACIs and EC along continuous tree cover and permafrost extent gradients (see Fig S7 and Fig R1), and (2) to understand the climatic, vegetation, and environmental controls on seasonal carbon cycles in the SEMs (see Fig 5). As for (1), we found a general consistency in trends of net CO_2 uptake from the ACIs and EC along tree cover and permafrost extent gradients (Fig S7 and Fig R1). For (2) we used %P as variables in the SEMs and found %P was negatively correlated with respiration, so that respiration is higher in warmer and less permafrost extensive regions (lines 231-270, description of SEM results).

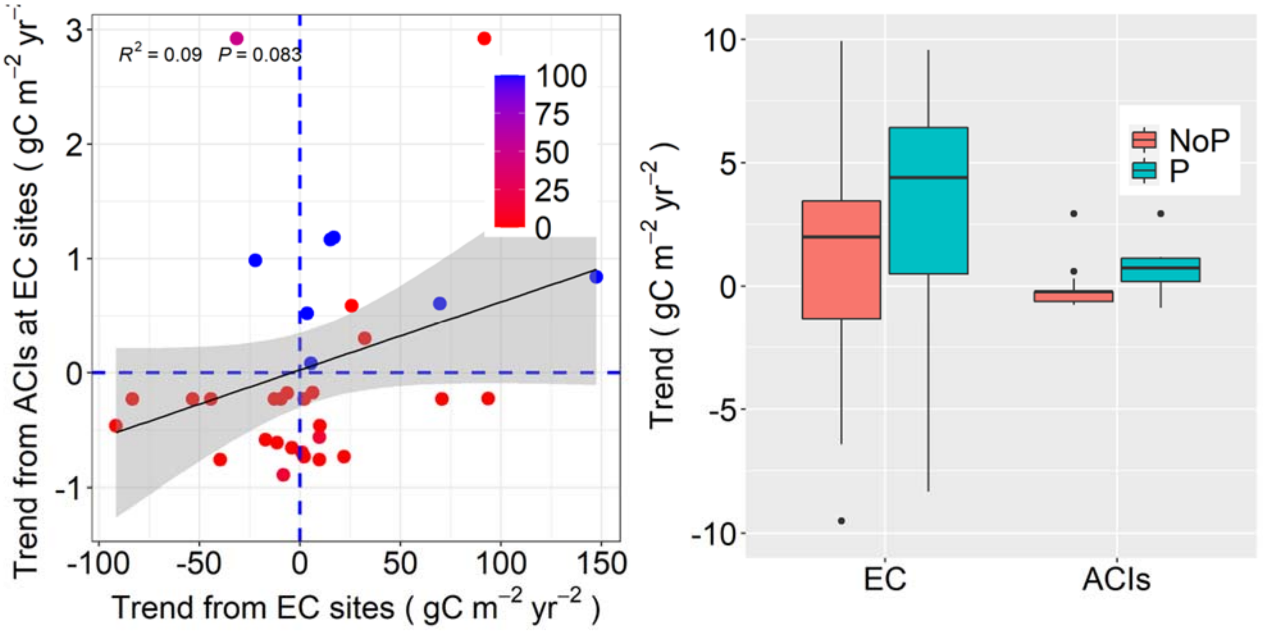

Fig. R1: Site-level comparison of trends of net CO_2 uptake using EC and ACIs at the EC site locations. Fig (a) showed the correlation between trends calculated from EC and ACIs at the EC site locations, colored by percent of permafrost extent. Fig (b) showed the average trends calculated from EC and ACIs at each EC site location averaged over permafrost and non-permafrost regions.

Regardless of whether the authors better estimate permafrost zonation, I believe trying to frame the results as a 'permafrost vs. non-permafrost' regional phenomenon creates a confusing

narrative that may the miss the actual mechanisms driving the documented variation in net CO2 uptake occurs. Specifically, I think the relative potential vegetation response to climate varies across the region and this is likely is the driver of the trend differences. Tundra shrubs often have a greater capacity to respond to climate than do tree species and this might be the reason for these results (see below).

Response: Thanks for the constructive suggestion. In this revision, we have reframed the analysis based on tree cover and permafrost. We found that tree cover has stronger controls on seasonal net CO_2 uptake than permafrost, suggesting a mechanistic link between vegetation and the carbon cycle as noted by the reviewer (lines 117-126 in main text).

Therefore, we have focused on understanding the variation of net CO_2 uptake along the vegetation gradient (Fig 1-4) in this revision. To reflect this change, we reported on how net CO_2 uptake varies along the tree cover gradient. We also added tree cover and permafrost as additional variables to explain the mechanisms underlying the seasonal dynamics of net CO_2 uptake in the SEM analysis in this revision, acknowledging their collinearity (lines 231-270, description of SEM results).

2) Vegetation cover: Typically in non-permafrost regions of the far northern high latitudes the dominant vegetation is continuous forest cover. Trees can extend from low latitudes (52-60 ºN to very high latitudes (60-70 ºN) in non-permafrost regions. However, in the so called 'permafrost zone' of this paper there is a more consistent transition that occurs from trees, to shrubs, to tundra (e.g. graminoid) across most of the studied areas. I expect that the strength of the C uptake response reflects this gradient more than anything related to permafrost presence (including NE Siberia where deciduous larch allows for a vigorous shrub presence). Shrub expansion both in terms of profile (height, density) and coverage influences snow dynamics in the shrub-to-tundra transitional areas. This feeds back onto the seasonality of flux dynamics as it influences snow accumulation and loss. This is really well-documented in the northern high latitudes (some of the authors have published on the topic).

Response: The reviewer is correct in that vegetation plays a more important role in regulating net CO_2 uptake. See our responses to previous comments and comments below.

3) Forest land use/disturbance: Using 52ºN latitude cutoff, then a significant fraction of the land cover in the non-permafrost is heavily affected by forest management (harvesting), and is more influenced by forest fires and insect epidemics (e.g. mountain pine beetle) relative to the non-permafrost areas. Some of it is mixed-agriculture and forest, again, unlike permafrost regions. I think the authors should address these issues in the manuscript or supplementary material.

Response: Thanks for the suggestions. We have considered forest management and fire disturbance, two of the most prevalent disturbance agents in the NHL, in the revised manuscript as summarized below.

1. For management, we used a newly derived forest management dataset for 2015 (Lesiv *et al.*, 2022), but did not find a significant relation between trends of net CO_2 uptake and forest management.

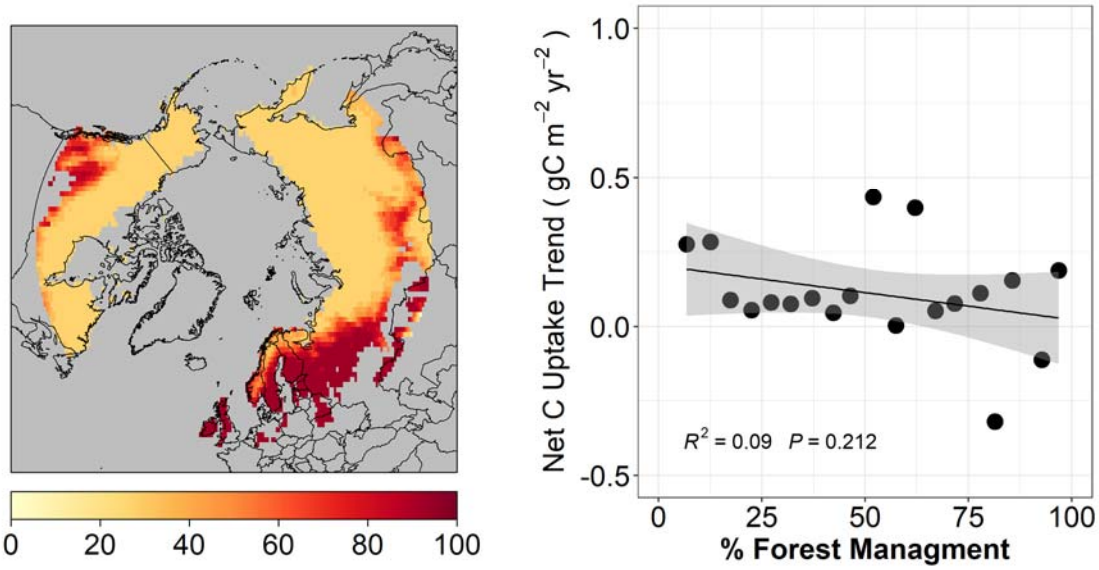

Fig. R2: Percent of forest management at 1-degree spatial resolution (left) and trend of net CO2 uptake along the percent of forest management gradient. Percent of forest management was calculated as percent of pixels with forest management activities divided by all forest pixels at 100-m resolution using data from (Lesiv *et al.*, 2022).

2. For fire disturbance, we found there is no NHL regional trend in burned area from 2001-2020 based on the MODIS burned area record (Fig R3, MCD64A1, V006). These results indicate that, fire disturbance is unlikely to significantly explain the regional trend in net CO_2 uptake, although many small fires (e.g. low intensity ground fires) are not detected by MODIS.

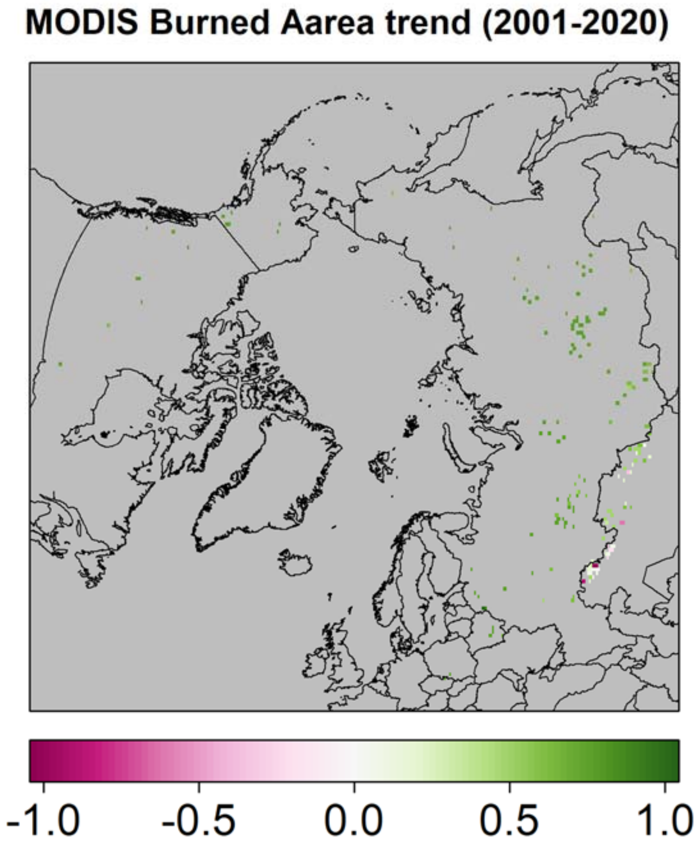

Fig. R3: Trend of burned area from 2001-2020 in the northern high latitude regions.

Potential solution
I think this article would be more informative if permafrost was treated as an independent variable in the SEM rather than a definitive category. The authors could assign potential percentage area of permafrost for different coverage (e.g., sporadic) grids based on permafrost distribution maps. The current framing effectively assumes that permafrost presence is continuous in all zones and causative, which is either inaccurate or disputable. If the authors could include vegetation cover type, maybe land use/disturbance (less important) and permafrost presence in their SEM modeling, then I think this would be an incredible paper. As it is currently framed, I think it is creating more confusion than clarity.
Response: Thank you for these excellent suggestions that greatly improved the quality and clarity of this work.

We have included percent tree cover (%TC) and permafrost extent, in addition to other climatic variables, in the SEM. We found %TC, together with Air T, are strong drivers of ecosystem productivity in the early growing season. This is consistent with t previous analyses indicating that temperature-controlled photosynthetic activity and increase in woody vegetation cover were among the major drivers of productivity and net CO_2 uptake in the early growing season. During the late growing season, we found %P has a negative influence on late growing season

respiration, suggesting warmer conditions have higher late growing season respiration. The net CO_2 uptake in the late growing season was primarily regulated by respiration, which was mainly controlled by increased labile carbon from enhanced early season productivity and temperature. please see lines 231-270 in main text for detailed description of results, and lines 153-175 in Materials and Methods for detailed description on data sources.

Minor concerns
There are a large number of spacing issues, very often in how units are displayed, and other small editorial issues. I did not go through and correct these.
Response: We have carefully checked the manuscript and have tried to eliminate these editorial issues.

Fig. 4. With a little movement of the numbers in the graph denoting the relationships, all of the correlation values could be fully visible.
Response: Suggestion adopted.

Reviewer #2 (Remarks to the Author):

This study analyzed multiple constraints on estimates of carbon cycling across the northern high-latitude region (>50 deg N) from atmospheric inversions, a terrestrial biosphere model ensemble, and flux tower observations and found an agreement in a trend of increasing annual net CO2 sink strength on land since the 1980s due to increasing uptake by plants outpacing increases in late season respiration.

General comments

This paper outlines a pretty extensive analysis of multiple data sources and models to diagnose and attribute trends in high latitude land carbon cycling. There is a mostly clear overall story, even if it deviates in various ways from the hypotheses and results of other studies. There are a lot of pieces packed into this manuscript, and generally the presentation of such could use some streamlining and clarity particularly with how this study's results may change (or not) the narrative developing from these other studies. Specific to this paper, though, there are many interesting results that come from some rather clever analyses that together will make a valuable contribution to the literature around these critical issues.

But again, there is so much happening here that it can be hard to follow the main storyline, especially when the onus is on the reader to go to the supplemental nearly every step of the way to find the relevant details of where this information comes from and how these conclusions were arrived at. I would recommend that, in a revised version, the authors consider places where the story could be streamlined while at the same time adding a few key details in particular

places so that there is enough explanation and justification in the main text so that the reader does not constantly have to refer to the supplemental material or try to interpret the incredible amount of information packed into the (25!) supplemental figures.
Response: Following the recommendation, we have focused on the most interesting results and streamlined the manuscript. To address this and other reviewer concerns, we made the following changes in the revised manuscript:

In this revision, our manuscript was restructured in a hypothesis-driven manner to address observed significant trends in net CO_2 uptake First, we determined and present the trends of net CO_2 uptake along the vegetation, permafrost and temperature gradient, and found tree cover has the strongest control on trends of net CO_2 uptake. Second, we explained the mechanisms underlying different net CO_2 uptake trends, by testing two hypotheses. Third, we used SEM to understand climate, vegetation and environmental controls on the seasonal carbon cycle processes. Finally, we discuss implications of the results, particularly in the context of understanding increasing carbon uptake and seasonal amplitude of net CO_2 in the northern high latitude region.

To focus on the main message of the manuscript, we moved some analyses into the appendix (e.g., temperature effects, Fig S14), and removed less pertinent analyses (e.g., moisture effects, change in freeze/thaw and snow conditions in previous version). We reduced the appendix figures from 25 to 14 to focus on the main message of the manuscript. To better reflect our main message, we also changed the title into "*Respiratory loss during late-growing season determines the net carbon dioxide sink along the tree cover-permafrost gradient in northern high latitude regions*".

Having said that, the flow of the investigation is logical, the analyses robust, and the methods defensible. It is just a lot of each!
Response: Thanks, and please see previous response.

Some general considerations with respect to interpreting the results:
-There's a lot of spatially variability and finer-scale heterogeneity in the continuous-discontinuous- sporadic-isolated permafrost gradient. It seems that those were lumped pretty broadly here, or was the finer-scale heterogeneity maintained?

Response: Please see response to R1

- How spatially-resolved are the atmospheric inversions with respect to their ability to separate fluxes among these regions?

Response: To understand the uncertainty in ACI estimates and their effects on trend estimates, we used the general linear mixed effects model (GLMM) to investigate the uncertainty in ACI

estimates from: (1) spread across different ACIs; (2) time-dependent differences in spread across ACI estimates; and (3) differences among ACIs in partitioning of fluxes across permafrost-vegetation gradients. GLMM results showed that even after considering multiple sources of uncertainty affecting the ACI estimates, significantly different trends of net CO_2 uptake were still observed across permafrost-vegetation gradients. Therefore, the ensemble mean of ACIs was able to separate fluxes among these regions. Please see *Supplementary text to describe in full detail about using mixed effects model analysis of net CO2 trends over permafrost and non-permafrost regions using ACIs* for full details (lines 741 – 832 in Materials and Methods).

In addition, we also conducted an extensive sensitivity analysis (see 3.1.3 Robustness analysis: for method details), which showed that the faster increasing rate of net CO_2 uptake over short-vegetated permafrost regions is robust. The results were briefly introduced in the main text (lines 151 – 167 in main text) as follows:

"We rule out several factors that potentially confound the observed increase in net CO2 uptake over short-vegetated permafrost regions by: (1) accounting for the uncertainties of ACI estimates from spread across ACIs, partitioning of fluxes between regions, and time-dependent differences in ACI spread (see supplementary text for full details); (2) assessing individual ACIs, where all of the inversions showed increasing net CO2 uptake in the permafrost region (Fig. S5); (3) conducting a sensitivity test on the time period considered (Fig. S6); and (4) showing that both large-scale patterns and site-level EC measurements had an increasing net CO2 uptake over permafrost regions, despite occurring at different rates due to scale mismatch between ACIs and EC footprints (Fig. S7); (5) assessing spatial and seasonal consistency of trends from individual ACIs (Fig. S8); and (6) confirming that the addition of more ACIs after 2000 did not alter the trends (Fig. S9 and S6). Therefore, despite generally large uncertainties among ACIs, our results are robust against outliers and in agreement with independent observations from EC data. Therefore, based on the most current atmospheric inversion estimates, the carbon sink strength of shrub and graminoid-dominated permafrost regions has been increasing significantly faster than tree-dominated non-permafrost regions in the NHL."

- How meaningful is the "non-permafrost" region that is arbitrarily >50 deg N but not in the permafrost zones? What about differences in PFT distributions occurring within and between permafrost vs. non-permafrost regions?

Response: The permafrost region (excluding permanent snow/ice and barren land), including sporadic (10%-50%), discontinuous (50%-90%), and continuous (>90%) permafrost, encompasses about 15.7 ×106 km_2, accounts for 57% of the NHL study area, and is dominated by tundra (shrubland and grass) and deciduous needleleaf (i.e., larch) forest that is regionally abundant in Siberia. The NHL non-permafrost region covers about 11.9 ×106 km2 and is dominated by mixed and evergreen needleleaf boreal forests. Please see Fig S1, and associated text.

- How consistent are the measurements and inversions over this long time period (1980-2017) in terms of methodology, especially the density and spatial distribution of observations (and comparing between early vs. later C balance estimates such as in lines 101-102)? Although I suppose all of this explanation is packed into supplementary text, as alluded to in lines 105 - 119 (which is a lot to digest, but…).
Response: The density of observations to constrain the ACIs is generally increasing over time, implying that the ACIs are generally becoming more accurate at higher spatial resolutions as more observations become available. In general, ACIs differ from each other mainly in their underlying atmospheric observations, transport models, spatial and temporal flux resolutions, land surface models used to predict prior fluxes, observation uncertainty and prior error assignment, and inversion methods. We have conducted a series analysis to make sure these factors do not affect the main conclusion of this analysis, including (1) using an ensemble mean of six different ACI products to better constrain the surface-to-atmosphere net ecosystem CO_2 exchange to reduced uncertainties from each individual ACI; and (2) conducting an extensive sensitivity analysis (see previous response).

Therefore, despite generally large uncertainties among ACIs, our trend results are found to be robust against outliers and in agreement with independent EC observations.

- And while this is touched on in sort of a hypothesis discussion near the end, the authors should consider more fully addressing the role (or lack thereof) that disturbances (particularly fire emissions and permafrost thaw) play in their study's results and especially interpreting them relative to the findings of other studies.
Response: See response to R1.

Specific comments
38, 42, 92, 99, 122-124, Figure 1, etc. Change yr-2 to yr-1?

Response: We used gC m^{-2} yr^{-1} to denote net CO^2 uptake, and gC m^{-2} yr^{-2} to denote the trend of net CO^2 uptake.

48 & 81. How is compensation(s) being used? Offsets? Balance? Fluxes?

Response: Thanks, we used *offset* for this instance.

67. Consider using AIMs, which seems more standard?

Response: Thanks for suggestion. We kept using ACIs in this revision.

76. Emerge

Response: Suggestion adopted.

79. were

Response: Suggestion adopted.

87. Note that it is ‘Materials and Methods’ section in the Supplemental Material document

Response: We have changed this to Materials and Methods.

91-97. Please state the time period for this analysis, along with the areas associated with each the permafrost and non-permafrost regions.

Response: We have stated clearly that it is between 1980-2017 in this revision.

93. As with the mean and trend, also include the value for the interannual variability?

Response: Thanks for the suggestion.

In this analysis, we calculate the contribution of interannual variability in NHL (F_{IAV}) following the method described in (Tagesson *et al.*, 2020). Basically, the F_{IAV} indicates fraction of global interannual variability contributed by the NHL.

154-156 “increasing CO_2 uptake in the EGS are similar between the non-permafrost and permafrost regions” (see also 179-180)

Response: Suggestion adopted.

189. This idea of “legacy effects” is interesting and worth thinking about and possibly exploring further. However, it is sort of just mentioned as a one-off thought here and not really explained - nor defined, i.e. how long of time periods did you look for these effects? Just within the season? Another potential longer-term effect that has been hypothesized is the stimulation of photosynthesis (in the following EGS, and in future years) when more N becomes available as a result of increased Rh in the LGS.

Response: Thanks for the suggestions. To simplify the message, this has been removed.

201-205 This translation of your results to recommendations for modelers is great!

Response: Thanks.

206 the acronym SEMs may not be needed here since it is not used again; however, it might be useful to provide a brief explanation of SEMs here, at least in the context of why and how you used the approach in this analysis.

Response: We have briefly introduced SEM in line 236 – 237 in the main text. "*SEM is a multivariate, hypothesis-driven method that is based on a structural model representing a hypothesis about the causal relations among interacting variables.*"

223-234 "in the LGS"

Response: Suggestion adopted.

227 re: 'net CO2 uptake in the LGS', I may be misinterpreting this but I was under the impression that there was net release in the LGS - just less than the magnitude of uptake in the EGS? (e.g. see lines 39-40, 157-158, 230)

Response: Your understanding is correct. We have clarified this

245-247 I understand that that there's a lot going on in this study and much of the detail is in the supplemental, but this is an example where it feel like at least a mention of the particular satellite system / data set used would be useful to include in the main text; e.g. 'SM' (soil moisture? Acronym not defined earlier. What data set was used? What NDVI data set was used? Time scale? Spatial resolution?)

Response: These contents have been removed to focus on the most interesting and relevant results in the revision.

247-248 'net uptake through respiration'?

Response: Suggestion adopted.

267 change 'indicates o' to "identifies"
Response: Suggestion adopted.

274 this statement may need just some context or justification, but the wording implies that permafrost regions are sources(?) that have 'switched' to sinks but it hasn't shown up in the observational record. That is not something that your results show (unless I am missing it?) i.e.

that they've always been sinks and have increased as such over your time period of analysis. Does that idea come from another study / studies? If so, when did this switch happen, relative to the time period of your analysis?

Response: These contents have been removed to focus on the most interesting and relevant results in the revision.

281-285 this part now seems to go the opposite way as you postulate that they could switch (again?) to sources due to fire, thaw, etc. But your results showing increased net uptake does not seem to suggest this, not to mention we assume (but not sure?) that the models may or may not handle fire, disturbance, thaw. This has been raised in many other studies, though, so perhaps citing some of those here and explaining how they do or do not jive with your results would be helpful here to clarify what you are trying to convey to the reader with these statements.

Response: Sorry for the confusion. In this paragraph, we intended to discuss the possible switch of CO_2 uptake status for the NHL forested regions. Our results have shown that net CO_2 uptake is increasing at a slower rate in NHL forested regions. As NHL forested regions are projected to be warmer and drier, the increasing respiratory CO_2 losses, together with the other carbon emissions processes (e.g., disturbance, moisture-stress induced productivity decline), may change the region into a CO_2 source. We have clarified this in the revision (lines 299-307 in the main text).

312-331 There are a lot of big ideas and concepts stuffed into each paragraph, even each sentence. Consider prioritizing some of these hypothesis, synthesizing a broader perspective, and/or adding some explanation or context where needed (e.g. 'SOC thermal coupling' - what does that mean and how does it relate to your study?)

Response: Thanks for the comments. In this revision, we only focused on discussing implications that are directly related to our results. To reflect this change, we merged these two paragraphs and highlight: (1) the importance of late-growing season respiration in affecting the carbon balance; (2) recommendation to DGVMs on how to better simulate late growing season respiration process (lines 329-352 in the main text).

319 should you still use the 'LGS' acronym here?

Response: we spell this out in the revision.

326 why are you focusing now on just the boreal forest?

Response: This has been removed.

332 does ‘as a whole’ mean geographically? and/or being comprehensive of all the budget and flux components (e.g. are you including fire in this statement?)

Response: geographically. We have classified this in the revision.

387. “...according to the ensemble of ACIs…”

Response: Suggestion adopted.

References cited in the response letter

Lesiv, M., Schepaschenko, D., Buchhorn, M., See, L., Dürauer, M., Georgieva, I., Jung, M., Hofhansl, F., Schulze, K., Bilous, A., Blyshchyk, V., Mukhortova, L., Brenes, C.L.M., Krivobokov, L., Ntie, S., Tsogt, K., Pietsch, S.A., Tikhonova, E., Kim, M., Di Fulvio, F., Su, Y.-F., Zadorozhniuk, R., Sirbu, F.S., Panging, K., Bilous, S., Kovalevskii, S.B., Kraxner, F., Rabia, A.H., Vasylyshyn, R., Ahmed, R., Diachuk, P., Kovalevskyi, S.S., Bungnamei, K., Bordoloi, K., Churilov, A., Vasylyshyn, O., Sahariah, D., Tertyshnyi, A.P., Saikia, A., Malek, Ž., Singha, K., Feshchenko, R., Prestele, R., Akhtar, I.u.H., Sharma, K., Domashovets, G., Spawn-Lee, S.A., Blyshchyk, O., Slyva, O., Ilkiv, M., Melnyk, O., Sliusarchuk, V., Karpuk, A., Terentiev, A., Bilous, V., Blyshchyk, K., Bilous, M., Bogovyk, N., Blyshchyk, I., Bartalev, S., Yatskov, M., Smets, B., Visconti, P., McCallum, I., Obersteiner, M. & Fritz, S. (2022) Global forest management data for 2015 at a 100 m resolution. *Scientific Data*, **9**, 199.

Tagesson, T., Schurgers, G., Horion, S., Ciais, P., Tian, F., Brandt, M., Ahlstrom, A., Wigneron, J.P., Ardo, J., Olin, S., Fan, L., Wu, Z. & Fensholt, R. (2020) Recent divergence in the contributions of tropical and boreal forests to the terrestrial carbon sink. *Nat Ecol Evol*, **4**, 202-209.

Reviewer comments, second round review:

Reviewer #1 (Remarks to the Author):

General comments
The new analysis makes this an important paper. The noteworthy contribution is the alignment of multiple streams of data and evaluation of permafrost and vegetation gradients alongside climatic trends. The methods are sound (albeit, I am only superficially familiar with ACI techniques).

But I believe the presentation has taken a step backward in terms of writing and this hurts the linkage between the presented data and conclusions. Figures have small mistakes and there are other editorial issues. I may not catch all of these and the authors should be able to make improvements with their own careful reading.

The first paragraph of the paper needs to be re-written. It does a poor job of introducing topics. Most important, toward the beginning of the paper you need to define what you mean by 'seasonal compensation' with a clear statement. This is not a common phrase.

One thing the authors should consider addressing is how regional gradients (%tree cover; %permafrost) that have developed over millennia reflect the potential response of these systems to a rapidly changing climate. By the end of the century, the arctic will have a climatic envelope that is completely novel relative to the lifetime of the vegetation that lives there; specifically, it is doubtful that tree cover will expand rapidly enough to affect what happens where tundra vegetation is currently located. For example looking at Figure 1f, there is a point in the 100% permafrost area that should be considered as a potential indicator of 'trouble'.

Related to this, the curvilinear patterns in Figure 1f and 4a suggest that the responses captured both by data and TRENDY models will not be straightforward across 'permafrost extent' and 'tree cover' for net co2 uptake. One thing that could be mentioned is that the places with mixtures of vegetation (~50% tree cover) seem to more rapidly increase net CO_2 uptake in response to climate change for TRENDY, possibly because the expansion potential from tree seed sources and growth of tree/shrub vegetation are dominant. I leave it to the discretion of the authors as to whether they want to make this point.

Specific comments
L50: impacts
L56-57: Are the responses of GPP and Respiration always asynchronous? I would qualify this statement.
L61: What is 'spatial compensation'? I know what you are trying to say but this sets up your entire paper and it needs a clearer statement.
L70-71: Tree cover extent is first introduced here. There needs to be an explanation earlier as to why different vegetation types, and permafrost, can have different effects on the asynchrony of GPP and RE.
L84: Exchange 'our' with 'us'
89-90: It is not clear what this means 'magnitude of seasonal net CO_2 uptake compensations'

L98: This needs to be rewritten 'late-growing season respiration-dominated CO_2 emissions'. A mouthful! What emissions would not be 'respiration-dominated'? Fire?
L111: You should have a citation for the 12% of land surface area and reference the supplemental material where you present global data.
L110-112: Related to the above, putting the results section/paragraph in the context of 'global' C uptake is strange way to begin. The paper is not about global C dynamics and you do not present the C balance of the entire globe anywhere. It is not even part of the introduction.
L122: 0.9 should be 0.90
L130: vagrant 's' in the text.
L144: Check this sentence.
L145: what is a 'yr-2?'….here and elsewhere?

L152-53: Not sure what this means "accounting for the uncertainties of ACI estimates from spread across ACIs"? Can this sentence be made more clear?

L205: vagrant 'r'

L285: Rewrite after 'vegetation cover,…'

L316-321: This sentence needs to be rewritten.

L354: This would be a good place to discuss the curvilinear nature of the relationships highlighted earlier.

Fig. 1. 'M' in mean annual temperature is cutoff.

Fig. 2. NDVI caption is bold.

Figure 2b and 2d. The histogram inserts are indecipherable. 'Y-2' for GPP

Fig. 3d. The equations could be moved downward to show the line/parameters better.

Check caption—Fig S13.

Reviewer #2 (Remarks to the Author):

Thank you for your thoughtful replies and explanations to all of the review comments. It was great to learn that your incorporation of permafrost and and tree cover as continuous variables improved the analysis, and that some of the other edits helped improve clarity of individual ideas as well as the overall message. This study will make an excellent contribution to the science and likely stimulate interesting follow-on studies ate various scales. Nice work.

REVIEWERS' COMMENTS

Reviewer #1 (Remarks to the Author):

General comments
The new analysis makes this an important paper. The noteworthy contribution is the alignment of multiple streams of data and evaluation of permafrost and vegetation gradients alongside climatic trends. The methods are sound (albeit, I am only superficially familiar with ACI techniques).
Response: Thank you for your constructive comments, that helped us to improve the quality and clarity of this work.

But I believe the presentation has taken a step backward in terms of writing and this hurts the linkage between the presented data and conclusions. Figures have small mistakes and there are other editorial issues. I may not catch all of these and the authors should be able to make improvements with their own careful reading.

Response: We have rewritten the first paragraph of Introduction following R1's suggestion and revised our manuscript carefully to eliminate the small mistakes and editorial issues. Please see our point-by-point response below.

The first paragraph of the paper needs to be re-written. It does a poor job of introducing topics. Most important, toward the beginning of the paper you need to define what you mean by 'seasonal compensation' with a clear statement. This is not a common phrase.
Response: Thank you for your suggestion. We have substantially revised our first paragraph and focused on introducing why seasonal change of net CO_2 uptake is important to understand two of the most significant change of carbon change in northern high latitude, including increasing terrestrial carbon sink and increasing amplitude of seasonal atmospheric CO_2 concentration over the past few decades. We believe this revision will lay a good foundation to our research questions. The first paragraph now reads "The northern high latitudes (NHL, > 50°N) are experiencing dramatic changes in carbon cycling, evidenced by an increase in the annual terrestrial net CO_2 uptake and in the amplitude of the seasonal cycles of atmospheric CO_2 over the past five decades [1-3], but the mechanisms underlying these changes remain highly uncertain. Net CO_2 uptake results from the imbalance between the much larger gross fluxes of plant photosynthesis and ecosystem respiration, which have asynchronous responses to seasonal climatic and environmental change [4-6]. For example, increased plant photosynthetic CO_2 fixation during the growing season [7,8] may be offset by enhanced respiratory CO_2 release in the fall and/or winter [9,10]. Such offsets in net CO_2 uptake among seasons (i.e., seasonal compensation) complicates the detection of the climate-carbon feedbacks at longer time scales over NHL ecosystems [4,11]. Further, the seasonal compensation in CO_2 uptake may vary among biomes given the different sensitivity of above- and belowground carbon cycle processes to climate and environmental controls over changing NHL permafrost conditions [12-14]. Therefore, understanding the magnitude, trends, and spatial patterns of seasonal net CO_2 uptake and its underlying mechanisms is important to address a fundamental question of whether net CO_2 exchange has changed over the NHL, especially in rapidly changing permafrost regions [15-17]. (lines 55-72)

”

Regarding the ‘seasonal compensation’, we have defined it as “offset in net CO_2 uptake among seasons” (lines 63-64).

One thing the authors should consider addressing is how regional gradients (%tree cover; %permafrost) that have developed over millennia reflect the potential response of these systems to a rapidly changing climate. By the end of the century, the arctic will have a climatic envelope that is completely novel relative to the lifetime of the vegetation that lives there; specifically, it is doubtful that tree cover will expand rapidly enough to affect what happens where tundra vegetation is currently located. For example looking at Figure 1f, there is a point in the 100% permafrost area that should be considered as a potential indicator of ‘trouble’. Related to this, the curvilinear patterns in Figure 1f and 4a suggest that the responses captured both by data and TRENDY models will not be straightforward across ‘permafrost extent’ and ‘tree cover’ for net co2 uptake. One thing that could be mentioned is that the places with mixtures of vegetation (~50% tree cover) seem to more rapidly increase net CO2 uptake in response to climate change for TRENDY, possibly because the expansion potential from tree seed sources and growth of tree/shrub vegetation are dominant. I leave it to the discretion of the authors as to whether they want to make this point.

Response: We appreciate this suggestion.

We have discussed the tree species migration and climate change and its potential implications on carbon cycle. Specifically, we have added the following text in the discussion (lines 283-286) “However, as tree migration will likely lag climate change, warming-induced species reassembly and permafrost degradation will significantly alter the above- vs belowground carbon cycle and its interaction [47], especially in climate-sensitive permafrost regions with intermediate tree cover [48].”

As for the curvilinear patterns in Figure 1f and 4a, we found rate of tree cover increase is higher at intermediate level of tree cover and permafrost extent (Fig R1, added as Fig S14 in this revision), which is consistent with study from (Scheffer *et al.*, 2012) that found dramatic shift in boreal tree cover will most likely occur in intermediate level of tree cover. We have highlighted this in discussion “but our results suggest that ecosystems underlaid by intermediate levels of permafrost extent may be a particularly strong contributor to enhanced net C uptake (Fig. 1f) partially due to higher rate of woody cover expansion (Fig S14). ” (lines 273-276)

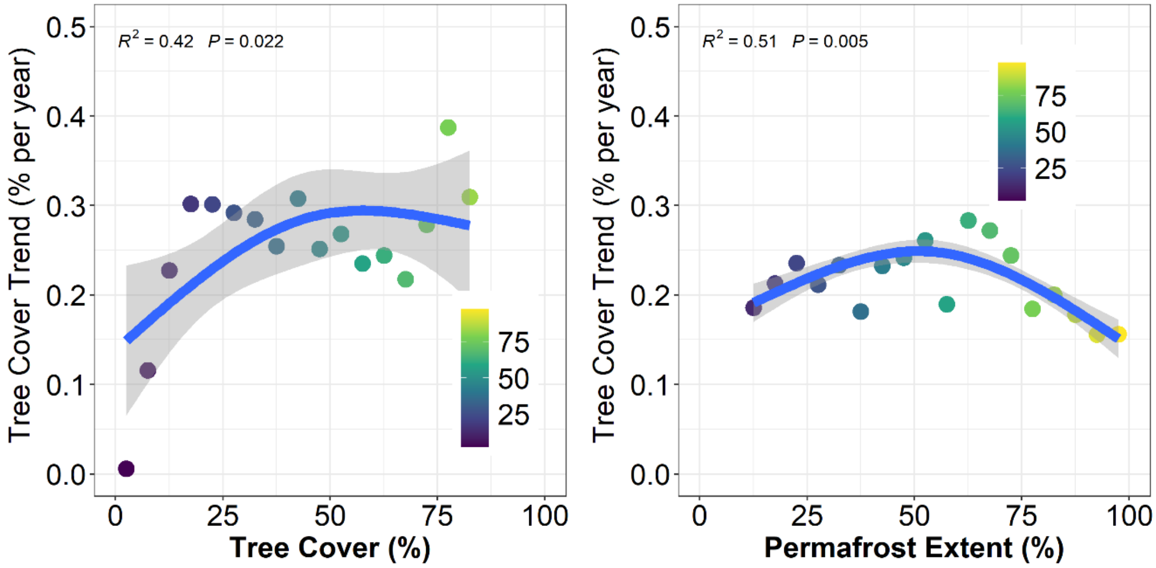

Fig. R1: Relationship between tree cover trend (% per year) and mean tree cover (%, left), and permafrost extent (%, right) at 5% intervals (from blue to yellow).

Specific comments
L50: impacts

Response: Suggestion adopted.

L56-57: Are the responses of GPP and Respiration always asynchronous? I would qualify this statement.

Response: Most studies have shown that GPP and Respiration respond differently to climate and environmental change.

L61: What is ‘spatial compensation’? I know what you are trying to say but this sets up your entire paper and it needs a clearer statement.

Response: This has been removed in this revision.

L70-71: Tree cover extent is first introduced here. There needs to be an explanation earlier as to why different vegetation types, and permafrost, can have different effects on the asynchrony of GPP and RE.

Response: This has been removed in this revision.

L84: Exchange ‘our’ with ‘us’

Response: Suggestion adopted.

89-90: It is not clear what this means 'magnitude of seasonal net CO2 uptake compensations'

Response: we defined this as "the degree to which seasonal net CO2 uptake is compensated by seasonal respiration losses" (line 82-83)

L98: This needs to be rewritten 'late-growing season respiration-dominated CO2 emissions'. A mouthful! What emissions would not be 'respiration-dominated'? Fire?

Response: This has been changed into "respiratory CO_2 loss during late-growing season".

L111: You should have a citation for the 12% of land surface area and reference the supplemental material where you present global data.

Response: This has been removed in this revision.

L110-112: Related to the above, putting the results section/paragraph in the context of 'global' C uptake is strange way to begin. The paper is not about global C dynamics and you do not present the C balance of the entire globe anywhere. It is not even part of the introduction.

Response: This has been removed in this revision.

L122: 0.9 should be 0.90

Response: Suggestion adopted.

L130: vagrant 's' in the text.

Response: Nice catch. This is corrected in this revision.

L144: Check this sentence.

Response: This has been changed.

L145: what is a 'yr-2?'….here and elsewhere?

Response: Since the unit for annual net CO_2 uptake (NCU) is gC $m^{-2}yr^{-1}$, the trend of NCU is the first derivative of annual NCU and has a unit of gC $m^{-2}yr^{-2}$.

L152-53: Not sure what this means "accounting for the uncertainties of ACI estimates from spread across ACIs"? Can this sentence be made more clear?

Response: Sorry for the confusion. We have changed this into "considering the uncertainties of ACI estimates resulting from variance across individual ACIs, partitioning of fluxes between regions, and time-dependent differences in ACI spread (supplementary text)". (lines 144-147)

L205: vagrant ‘r’

Response: This is corrected in this revision.

L285: Rewrite after ‘vegetation cover,…’

Response: Suggestion adopted.

L316-321: This sentence needs to be rewritten.

Response: This sentence has been revised to improve its clarity.

L354: This would be a good place to discuss the curvilinear nature of the relationships highlighted earlier.

Response: we have discussed earlier in the discussion section. see previous response.

Fig. 1. ‘M’ in mean annual temperature is cutoff.

Fig. 2. NDVI caption is bold.

Figure 2b and 2d. The histogram inserts are indecipherable. ‘Y-2’ for GPP

Fig. 3d. The equations could be moved downward to show the line/parameters better.

Check caption—Fig S13.

Response: All these minor issues are fixed. Please see revised figures for modifications.

Reviewer #2 (Remarks to the Author):

Thank you for your thoughtful replies and explanations to all of the review comments. It was great to learn that your incorporation of permafrost and and tree cover as continuous variables improved the analysis, and that some of the other edits helped improve clarity of individual ideas as well as the overall message. This study will make an excellent contribution to the science and likely stimulate interesting follow-on studies ate various scales. Nice work.

Response: Thank you for your comments.
